# Transcriptional analysis and histochemistry reveal that hypersensitive cell death and H_2_O_2_ have crucial roles in the resistance of tea plant (*Camellia sinensis* (L.) O. Kuntze) to anthracnose

**DOI:** 10.1038/s41438-018-0025-2

**Published:** 2018-04-01

**Authors:** Yuchun Wang, Xinyuan Hao, Qinhua Lu, Lu Wang, Wenjun Qian, Nana Li, Changqing Ding, Xinchao Wang, Yajun Yang

**Affiliations:** Tea Research Institute, Chinese Academy of Agricultural Sciences/National Center for Tea Improvement/Key Laboratory of Tea Biology and Resources Utilization, Ministry of Agriculture, Hangzhou, 310008 People’s Republic of China

## Abstract

Anthracnose causes severe losses of tea production in China. Although genes and biological processes involved in anthracnose resistance have been reported in other plants, the molecular response to anthracnose in tea plant is unknown. We used the susceptible tea cultivar Longjing 43 and the resistant cultivar Zhongcha 108 as materials and compared transcriptome changes in the leaves of both cultivars following *Colletotrichum fructicola* inoculation. In all, 9015 and 8624 genes were differentially expressed between the resistant and susceptible cultivars and their controls (0 h), respectively. In both cultivars, the differentially expressed genes (DEGs) were enriched in 215 pathways, including responses to sugar metabolism, phytohormones, reactive oxygen species (ROS), biotic stimuli and signalling, transmembrane transporter activity, protease activity and signalling receptor activity, but DEG expression levels were higher in Zhongcha 108 than in Longjing 43. Moreover, functional enrichment analysis of the DEGs showed that hydrogen peroxide (H_2_O_2_) metabolism, cell death, secondary metabolism, and carbohydrate metabolism are involved in the defence of Zhongcha 108, and 88 key genes were identified. Protein–protein interaction (PPI) network demonstrated that putative mitogen-activated protein kinase (MAPK) cascades are activated by resistance (R) genes and mediate downstream defence responses. Histochemical analysis subsequently validated the strong hypersensitive response (HR) and H_2_O_2_ accumulation that occurred around the hyphal infection sites in Zhongcha 108. Overall, our results indicate that the HR and H_2_O_2_ are critical mechanisms in tea plant defence against anthracnose and may be activated by R genes via MAPK cascades.

## Plant defence: pathways for tea protection

A comparison of gene activity in resistant and susceptible tea cultivars provides insights into mechanisms of defence against one of the most devastating tea diseases: anthracnose. Tea (*Camellia sinensis*) is an important crop in many tropical countries; however, yields can be devastated by the fungus *Colletotrichum*, the causal agent of anthracnose. Xinchao Wang and Yajun Yang, of the Chinese Academy of Sciences’ Tea Research Institute in Hangzhou, explored the defence response in tea cultivars infected with *Colletotrichum*. Discrepancies in the genes activated in resistant and susceptible cultivars suggest resistance is mediated by specific molecular signalling pathways, known resistance (R) genes, and involves the production of hydrogen peroxide and initiation of programmed cell death around sites of infection. These mechanisms could be harnessed to develop tea cultivars with greater resistance to anthracnose.

## Introduction

Tea plant (*Camellia sinensis* (L.) O. Kuntze) is a perennial evergreen woody plant that is widespread throughout tropical and subtropical areas, such as China, India, Kenya and Sri Lanka. As an important commercial product, the fresh shoots of tea plant provide an wide variety of nutrition for the human body, including flavonoids, alkaloids and theanine. Long-term tea drinking can protect against different diseases; therefore, tea has become the most popular healthy, non-alcoholic beverage in the world^1,2^. However, tea plant is frequently affected by many kinds of disease. Of these diseases, anthracnose, which is caused by *Colletotrichum*, is one of the most devastating diseases to tea plant^3^. *Colletotrichum* damages mature tea plant leaves, affecting the growth and yield of the plant^4^.

Host plants have evolved various defence mechanisms during interactions with plant pathogens. The first type involves pathogen- or microbial-associated molecular pattern (PAMP or MAPM, respectively)-triggered immunity (PTI)^5^. Pattern recognition receptors (PRRs) located on host plasma membranes recognise PAMPs or MAMPs, and these complexes induce mitogen-activated protein kinases (MAPKs) and/or calcium signalling; these MAPKs and/or calcium signalling trigger a series of defence responses, which results in the suppression of pathogen colonisation^6^. However, for successful invasion, adapted pathogens have evolved numerous virulence proteins called effectors to suppress or escape PTI in order to achieve infection. In turn, hosts also have evolved genes that encode intracellular nucleotide-binding site leucine-rich repeat (NBS-LRR) proteins that can specifically recognise effectors; this recognition activates a second host immune response named effector-triggered immunity (ETI) to restrict pathogen growth^7,8^. Interestingly, ETI is a faster and stronger version of PTI, and usually regulates the generation of host programmed cell death (PCD) in addition to the production of reactive oxygen species (ROS) at the site of pathogen infection^9^.

*Colletotrichum* is one of the largest genera of pathogens. *Colletotrichum* species are pathogenic to more than 3200 plants and cause large economic losses^10^. The hypersensitive response (HR) is a phenomenon of PCD. The HR is one of the most well-known resistance reactions and is associated with host resistance to *Colletotrichum*. For example, O’Connell et al.^11^ compared differences in resistance reactions between incompatible and compatible Arabidopsis plants in response to *Colletotrichum destructivum* at different infection phases, the results demonstrated that the incompatible Arabidopsis plants produced a rapid HR and deposited both callose and papillae in the infected epidermal cells following *C. destructivum* inoculation. In general, generation of the HR is associated with the activation of NBS-LRRs by pathogen effectors, the NBS-LRRs act together with multiple defence-related genes in plants to defend against *Colletotrichum*^9,12,13^. The resistance mechanism of tea plants has been loosely explained based on the results of several studies. For example, by studying tea plant defence to grey blight disease caused by *Pestalotiopsis* species, Senthilkumar et al.^14^, who used suppressive subtractive hybridisation techniques, suggested that the HR and ROS play crucial roles in tea plant resistance to *P. theae*. Moreover, Palanisamy and Mandal^15^ reported that the antioxidative enzymes associated with ROS in resistant tea cultivars have higher activity than those in susceptible cultivars following *Pestalotiopsis* sp. infection. Another important leaf disease of tea plant is blister blight, which is caused by *Exobasidium*. The results of Jayaswall et al.^16^ based on transcriptome analysis indicated that numerous defence-related genes are upregulated upon induction by *Exobasidium vexans* in tea plant, and a great number of well-known NBS-LRR genes are involved in the defence response. Despite many studies on tea plant resistance to pathogens, information on the molecular mechanism of resistance against *Colletotrichum* in tea plant is poorly understood.

To elucidate the resistance mechanism of tea plant to anthracnose, we previously used conidial suspensions of *Colletotrichum* to inoculate resistant (cultivar Zhongcha 108, ZC108) and susceptible (cultivar Longjing 43, LJ43) *Ca. sinensis* cultivars. ZC108 was produced by irradiating the offspring of LJ43. We speculated that the resistance mechanism of tea plant may be associated with NBS-LRR genes as well as the phenylpropanoid and flavonoid pathways based on our previous results using microarray data^17^. At the same time, we proved that flavonoid and caffeine biosynthesis are involved in tea plant defence to *Colletotrichum fructicola* infection^4^. Therefore, in the present study, to further reveal the resistance mechanism of tea plants to anthracnose, we comparatively analysed the changes in transcription levels in the leaves of both cultivars following *C. fructicola* inoculation using RNA-sequencing (RNA-Seq) and revealed a possible resistance mechanism in the tea plant response against anthracnose.

## Materials and Methods

### Plant material, fungal isolates and treatment

Tea plants (*Camellia sinensis* (L.) O. Kuntze) of the resistant cultivar ZC108 and the susceptible cultivar LJ43 as well as isolates of *C. fructicola* L33 were maintained as described previously^4,17^. Three-year-old plants were used as experimental materials. The plants were maintained in the glasshouse (28 °C, 14 h light, 80% humidity) and inoculated with conidial suspensions of *C. fructicola* (10^6^ spores/mL). For pathogenicity tests, wound inoculation of plants was performed in vitro based on the method described by Wang et al.^17^. The third leaves of ZC108 and LJ43 were sampled at 3, 7 and 11 days post inoculation (dpi). The non-wound inoculation of plants in vivo was used for RNA-Seq analysis as described by Jayaswall et al.^16^. Leaf tissues (first-fourth leaves) of ZC108 and LJ43 were sampled at 0 h (before inoculation), 24 and 72 h post inoculation (hpi).

### RNA isolation, library construction and sequencing

A total of 18 RNA samples were isolated using the cetyl-trimethylammonium bromide method described by Hao et al.^18^. The RNA quality was verified by a 1% denaturing agarose gel and a NanoDrop 2000 system (Thermo Scientific, Delaware, USA). Total RNA was used to construct cDNA libraries using a TruSeq RNA Sample Prep Kit (Illumina, San Diego, USA) according to the manufacturer’s protocol. The cDNA library was sequenced on an Illumina HiSeq^TM^ 2000 platform, and paired-end reads in 150 bp length were yielded.

### Data analysis

The detailed processes of de novo assembly, functional annotation and differentially expressed gene (DEG) identification were performed in accordance with the methods of Hao et al.^18^. Briefly, de novo assembly was performed using the Trinity (v2.2.0) programme^19^. By BlastX analysis (Basic Local Alignment Tool (BLAST) 2.2.30+) with the non-redundant (NR) database and TAIR database (Athaliana_167_TAIR10.protein.fa and Athaliana_167_TAIR10. annotation_info.txt) (http://www.arabidopsis.org/), the best hits (with a significant *E*-value of <1e^−5^) were assigned to the assembled transcripts of tea plant. RSEM v1.2.11 programme was used to analyse the RNA-Seq data for alignment and expression calculation^20^. The expression patterns and posterior probability of differential expression ‘posterior probability of differential expression’ (PPDE) values of each gene/contig were estimated by EBSeq v1.1.5 ^21^, and the DEGs were identified with PPDE = 1. The RNA-Seq raw data have been deposited in the NCBI Sequencing Read Archive database and can be accessed with the following accession numbers: SRR5986350; SRR5986349; SRR5986352; SRR5986351; SRR5986337; SRR5986353; SRR5986348; SRR5986347; SRR5986346; SRR5986345; SRR5986343; SRR5986339; SRR5986340; SRR5986338; SRR5986342; SRR5986344; SRR5986354; and SRR5986341. A Venn diagram was constructed using software that is available online (http://bioinformatics.psb.ugent.be/webtools/Venn/). Gene Ontology (GO) term enrichment was analysed by Gene Ontology Enrichment Analysis Software Toolkit (GOEAST), and statistical enrichment was considered when *P* < 0.05^22^. The Kyoto Encyclopedia of Genes and Genomes (KEGG) analysis was performed by the Database for Annotation, Visualisation and Integrated Discovery^23^. The short time-series expression miner software (STEM) software was used for analysing the different patterns of the shared DEG expression in both the resistant and susceptible tea plant cultivars^24^. Besides, the protein–protein interaction (PPI) network was constructed based on the data produced by the Search Tool for the Retrieval of Interacting Genes database (http://string-db.org/) and visualised using Cytoscape software (version 3.5.1)^25^.

### Microscopic observations

The leaves were collected at 12, 24, 48, 72 and 96 hpi and were cut into 0.5 cm segments, which were then used for observation of hypersensitive cell death and hydrogen peroxide (H_2_O_2_) accumulation. Trypan blue (Sangon Biotech Company, Shanghai, China) was used for the detection of hypersensitive cell death, as described by Faoro et al.^26^. The segments were boiled for 5 min in 1 mg/mL trypan blue solution (phenol:lactic acid:glycerol:distilled water = 1:1:1:1), after which the tissues were decolourized by chloral hydrate (Sangon Biotech Company, Shanghai, China). Diaminobenzidine (DAB) (Sangon Biotech Company, Shanghai, China) was used to visualise H_2_O_2_ accumulation in accordance with the method of Hao et al.^27^. The segments were infiltrated with 1 mg/mL DAB solution (pH = 3.8) and were vacuum-infiltrated for 10 min. After 12 h, the segments were cleared in saturated chloral hydrate and then discoloured in boiling 95% ethanol. Above treated samples were mounted on glass slides in 50% glycerol, and were observed under a Nikon 80i microscope (Japan).

### Gene expression validation by quantitative real-time PCR (qRT-PCR)

For qRT-PCR, 1 µg of total RNA used in the previous RNA-Seq library construction was used for cDNA synthesis. A PrimeScript RT enzyme with a gDNA eraser (Takara, Japan) was used for cDNA synthesis. qRT-PCR was performed on an Applied Biosystems 7500 Sequence Detection System using SYBR Premix Ex Taq™ II (Takara, Japan). The primers in this step are listed in Supplementary Table S1. The polypyrimidine tract-binding protein (*CsPTB1*) gene was used as an internal control^28^. The relative expression levels were calculated using the 2^−ΔΔCt^ method^29^.

## Results

### Pathogenicity tests

**Fig. 1:**
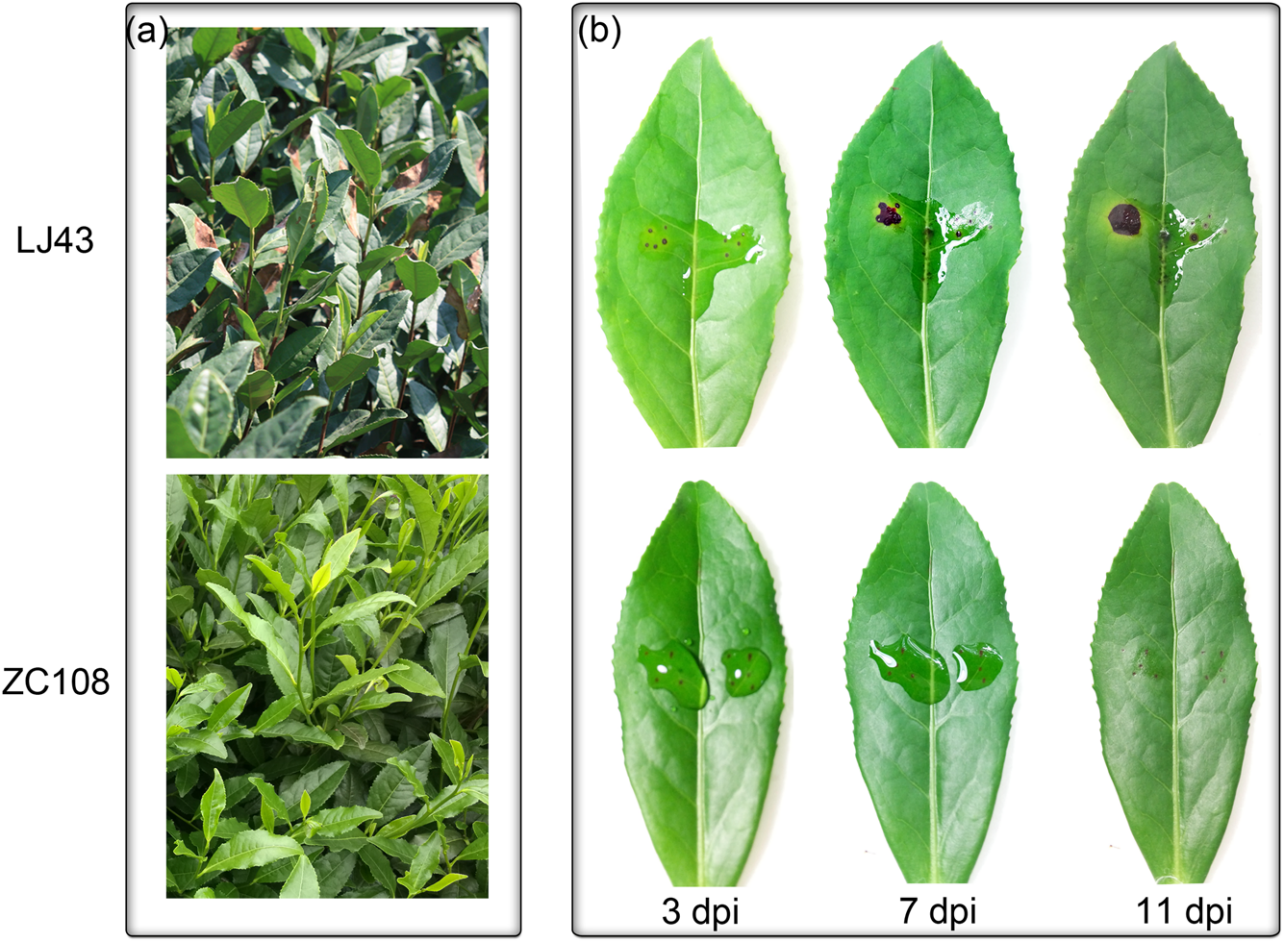
Altered disease resistance of resistant (ZC108) and susceptible (LJ43) tea plant leaves to anthracnose. **a** The images were taken from the same areas under natural conditions; **b** differential disease resistance of LJ43 and ZC108 leaves to *C. fructicola* infection in vitro

The resistant cultivar ZC108 is significantly more resistant to anthracnose in the field than is the susceptible cultivar LJ43 (Fig. 1a). To confirm these results, we inoculated the wounded mature leaves of ZC108 and LJ43 in vitro with conidial suspensions of *C. fructicola* (10^6^ spores/mL). Pathogenicity tests showed that LJ43 leaves displayed the typical brown lesions of anthracnose disease around wounded areas during pathogen infection (3, 7 and 11 dpi). However, the ZC108 leaves did not exhibit clear disease symptoms during inoculation (Fig. 1b). The in vitro results were consistent with the field results.

### Sequencing, assembly and DEG identification

In total, 878 273 717 raw data reads were generated from 18 samples. After the raw read sequences were filtered and passed through quality control, 851 249 406 clean data were obtained (Supplementary Table S2). Based on the high-quality clean data, a total of 864 790 transcripts were assembled across all 18 samples. After their de novo assembly by the Trinity (version 2.2.0) pipeline, the transcripts had an average length of 611 bp and an N50 of 788 (Supplementary Table S3). A total of 497 332 unigenes were generated, of which 234 496 and 134 208 were annotated by BLAST analysis using the NR database and The Arabidopsis Information Resource (TAIR10) according to significant hits (*E*-value < 1e^−5^), respectively (Supplementary Table S3). All unigenes were grouped into 203 expression patterns (Supplementary Table S4). Total of 49 784 and 49 661 DEGs were detected in the resistant and susceptible cultivars, respectively, following the elimination of genes that were not differentially expressed (Supplementary Table S4). In our study, the significant DEGs annotated by TAIR10 (*E*-value < 1e^−5^) were used to further analyse their function in both cultivars in response to *C. fructicola*. Total of 9015 and 8624 DEGs were identified in the inoculated leaves of ZC108 and LJ43, respectively (PPDE = 1; Supplementary Fig. S1). Compared with the unigene transcription at 0 h (control), at 24 hpi, 4581 DEGs were upregulated and 4254 were downregulated in ZC108; however, 4825 DEGs were upregulated and 3590 were downregulated in LJ43; at 72 hpi, the number of upregulated DEGs reached 5945 in ZC108, and 3058 DEGs were downregulated; in LJ43, 4554 upregulated and 4051 downregulated DEGs were identified (Supplementary Fig. S1). To identify changes in the resistance mechanism of *Ca. sinensis* during the *C. fructicola* infection progress, the DEGs of ZC108 and LJ43 annotated by the Arabidopsis database were used for the further analysis. Relevant information on the DEGs is listed in Supplementary Table S5.

### Investigation of DEGs and pathways involved in both resistance and susceptible cultivars

**Fig. 2:**
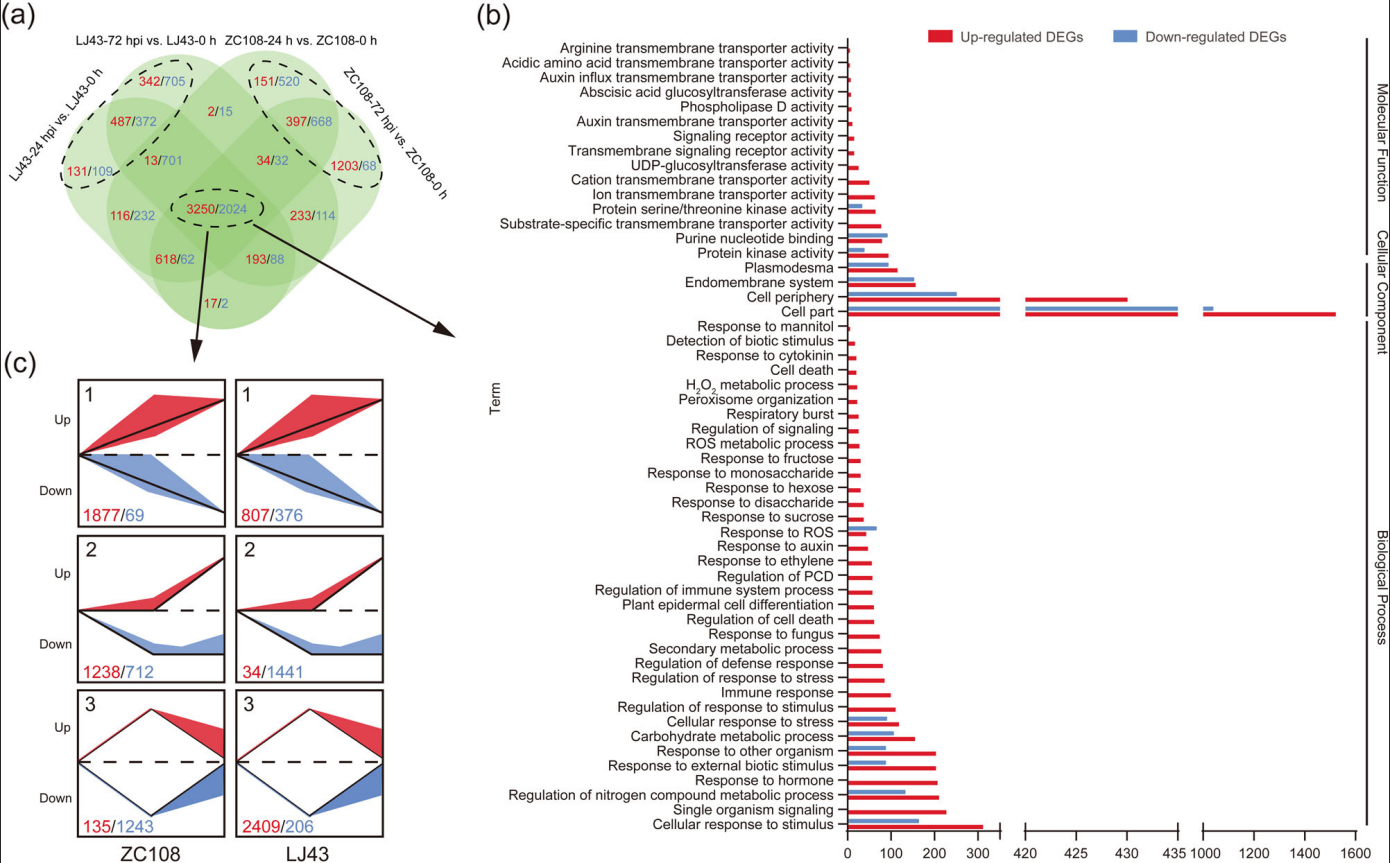
Shared genes in both resistant and susceptible *Ca. sinensis* leaves. **a** Venn diagram of the up- and downregulated DEGs in ZC108 and LJ43 leaves at 24 and 72 hpi, respectively; **b** GO functional classification of the 3250 up- and 2024 downregulated shared genes in both ZC108 and LJ43 leaves; **c** DEG patterns of the shared genes in both the ZC108 and LJ43 leaves. The cluster analysis was performed using the STEM Clustering method; the top number indicates the profile ID number, and the bottom number indicates the number of genes. The number of up- and downregulated DEGs is shown in red and blue, respectively

To identify the resistance mechanisms in response to anthracnose in both the resistant and susceptible tea cultivars, all the DEGs were assessed in the two cultivars at 24 and 72 hpi. The Venn diagram illustrated that the 3250 up- and 2024 downregulated DEGs in both ZC108 and LJ43 leaves are involved in the response to *C. fructicola* during the infection progress (Fig. 2a). Based on the GO analysis, these DEGs were enriched in 216 terms, including 176 biological process (the upregulated DEGs involved in 111 pathways and downregulated DEGs in 102 pathways), 8 cellular component (the upregulated DEGs involved in 4 pathways and downregulated DEGs in 8 pathways) and 32 molecular function (the upregulated DEGs involved in 25 pathways and downregulated DEGs in 16 pathways); among them, the upregulated DEGs were mainly enriched in the functional pathway that were mostly associated with disease resistance, such as the response to sugar metabolism (sucrose, disaccharides, hexose, monosaccharides, fructose and mannitol), phytohormones (ethylene, auxin and cytokinin), ROS, biotic stimulus and signalling, transmembrane transporter activity, protease activity and signalling receptor activity (Fig. 2b, the complete results of the GO enrichment are listed in Supplementary Fig. S2). Using the STEM, the 3250 up- and 2024 downregulated shared DEGs were clustered into 3 profiles (Fig. 2c). Each profile represents a group of genes that exhibit similar expression trends. For the upregulated DEGs, profile 1 had the most genes (1877), followed by profiles 2 (1238) and 3 (135) in the resistant cultivar ZC108; in contrast, the most genes in the susceptible cultivar LJ43 were classified into profile 3 (2409), followed by profiles 1 (807) and 2 (34). More genes whose expression continuously increased (profiles 1 and 2) were identified in ZC108 (3115) than in LJ43 (841). For the downregulated DEGs, profile 3 had the most genes (1243), followed by profiles 2 (712) and 1 (69) in the ZC108; correspondingly, the most genes were enriched in profile 2 (1441) in the LJ43, and the profile 3 was least enriched in the number of genes (206). In addition, 35 up- and 25 downregulated DEGs associated with disease resistance were observed during the infection process; the fold changes of these genes were markedly greater in ZC108 than in LJ43 (Supplementary Table S6). Together, various types of defence in both the resistant and susceptible tea plant cultivars were involved in the response to anthracnose, but the effectiveness of the expression of the shared genes in the resistant cultivar was better than that in the susceptible cultivar.

### Identification of specific pathways in resistant cultivars

**Fig. 3:**
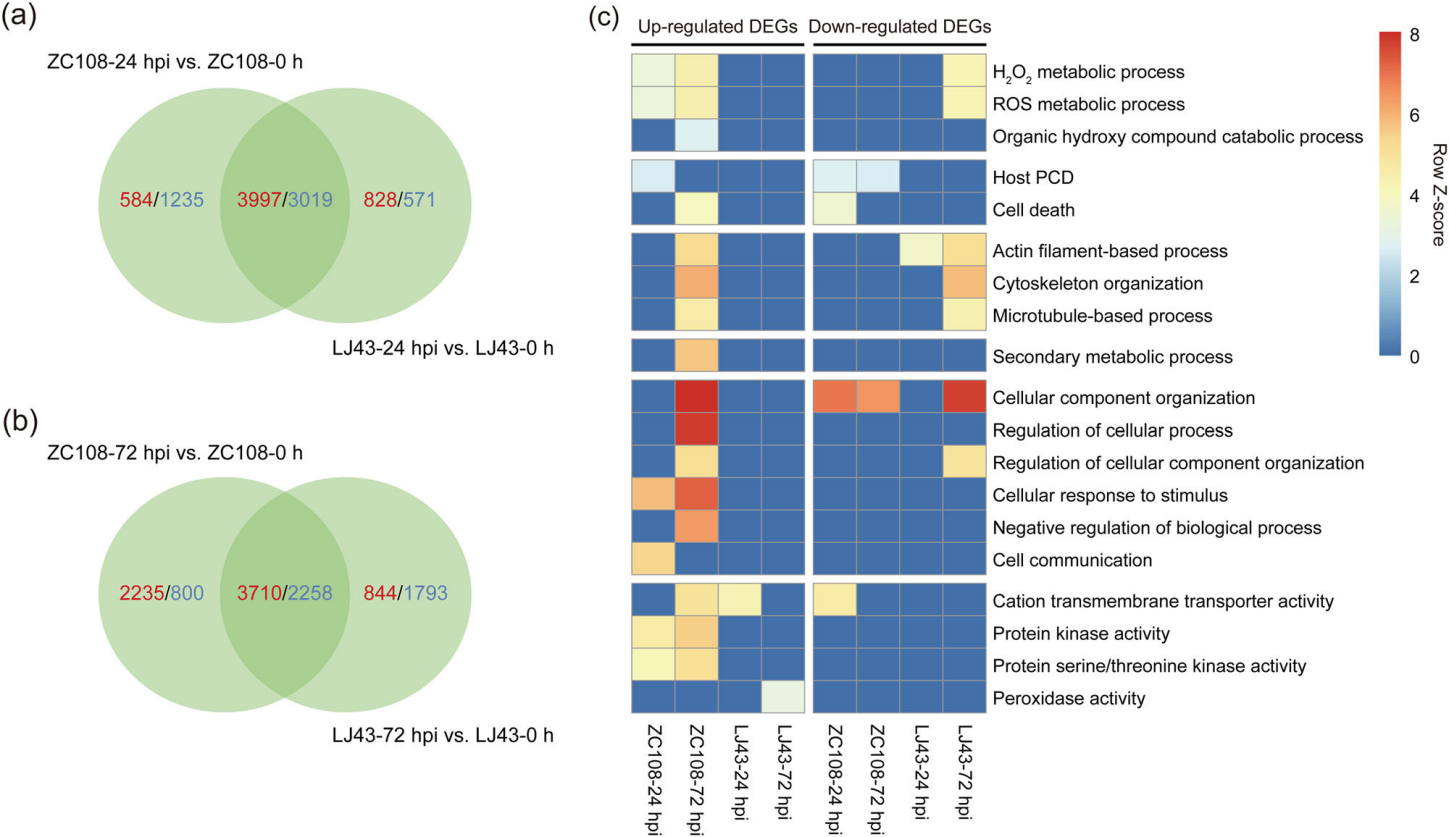
Change in DEGs in resistant (ZC108) and susceptible (LJ43) *Ca. sinensis* cultivars inoculated with *C. fructicola* during the infection process in comparison with their respective controls (0 h). **a**, **b** Venn diagram of the DEGs in ZC108 and LJ43 leaves at 24 and 72 hpi, respectively; **c** heatmap of DEGs specific to ZC108 and their respective homologous genes in LJ43 and of enriched GO terms related to disease resistance identified from DEGs. Terms are considered enriched when *P* < 0.05

The Venn diagram of the up- and downregulated DEG data of both cultivars shows that 584 and 828 upregulated DEGs as well as 1235 and 571 downregulated DEGs were specifically expressed at 24 hpi in ZC108 and LJ43, respectively (Fig. 3a). More specific upregulated DEGs were identified in ZC108 (2235 DEGs) than in LJ43 (844 DEGs) at 72 hpi, while the number of specific downregulated DEGs decreased in the ZC108 (800) dramatically, reversely increased in the LJ43 (1793; Fig. 3b). The GO analysis indicated that these DEGs were enriched in multiple pathways associated with disease resistance. The number of DEGs clearly changed during the infection process (*P* < 0.05; Supplementary Fig. S3). In particular, DEGs were enriched in ROS (H_2_O_2_ metabolic process, organic hydroxyl compound catabolic process and ROS metabolic process), cell death (host PCD and cell death), cytoskeleton (cytoskeleton organisation, actin filament-based process and microtubule-based process), secondary metabolic process, regulation of biological process (regulation of catabolic process, regulation of cellular process, regulation of cellular component organization, negative regulation of biological process, cellular response to stimulus and cellular component organization) and protease activity (protein kinase activity, protein serine/threonine kinase activity, cation transmembrane transporter activity and peroxidase activity) in ZC108, and the number of DEGs significantly increased during *C. fructicola* infection. In contrast, these DEGs were not enriched in the susceptible cultivar, and the number of DEGs decreased (Fig. 3c). Taken together, the results revealed that cell death and ROS play important roles in tea plant defence to anthracnose.

### Specific DEGs identified at different times after inoculation in resistant cultivars

**Fig. 4:**
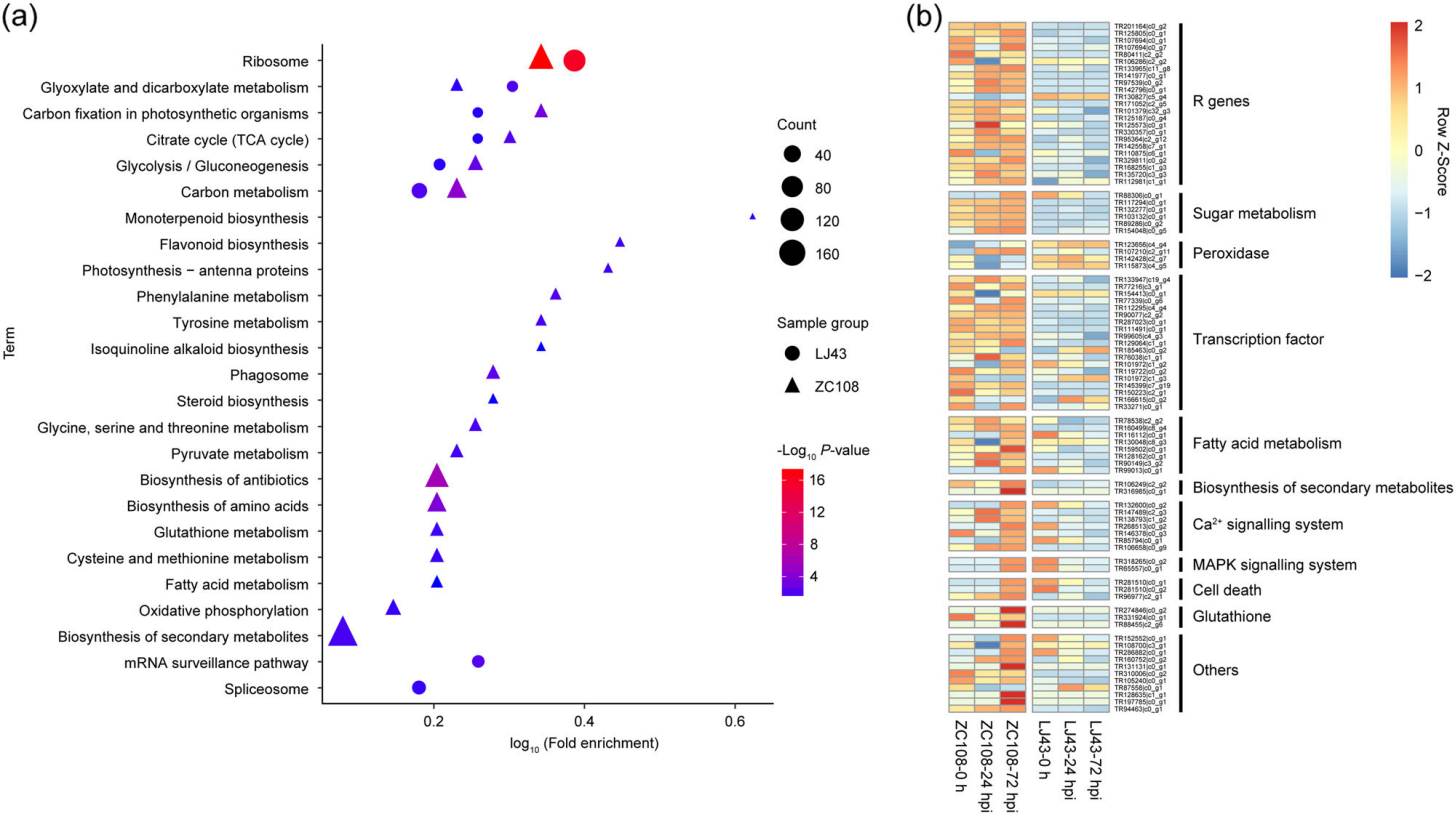
DEGs specific to resistant *Ca. sinensis* inoculated with *C. fructicola*. **a** KEGG classifications of the 3007 and 2146 DEGs specific to resistant and susceptible *Ca. sinensis* inoculated with *C. fructicola*; **b** heatmap of 88 key DEGs expressed specifically in the resistant *Ca. sinensis* cultivar

The data in Fig. 2a demonstrated that the 3007 DEGs (1751 upregulated and 1256 downregulated) and the 2146 DEGs (960 upregulated and 1186 downregulated) specific to the resistant and the susceptible *Ca. sinensis* cultivar were induced by the *C. fructicola* infection process, respectively. To identify the function of these DEGs, the KEGG analysis was used. In the susceptible *Ca. sinensis* cultivar, 2146 DEGs were only enriched in 8 pathways; comparatively, 3007 DEGs in the resistant cultivar were enriched in 23 pathways, of which flavonoid biosynthesis and phenylalanine metabolism were markedly enriched (*P* < 0.01 and fold enrichment < 0.4; Fig. 4a), suggesting that these two pathways play important roles in tea plant defence against anthracnose. Among the 3007 DEGs, 88 key genes associated with disease resistance were identified, including genes involved with pathogen receptors (23); peroxidase (4); signalling transduction, including Ca^2+^ signalling (7) and MAPK signalling systems (2); sugar metabolism (6); secondary metabolites (2); fatty acids (8); glutathione (3); transcription factors (TFs) (19); cell death (3); wall-associated kinases (2); protein kinases (1); and cytochrome P450 (3), as well as other defence function genes (5). The expression of all these genes were significantly changed in ZC108, but no change or opposite expression patterns were observed in LJ43 during *C. fructicola* infection (Fig. 4b). Information of the 88 key genes associated with disease resistance were listed in Supplementary Table S7.

### Regulatory network analysis of selected genes involved in the resistance response

**Fig. 5:**
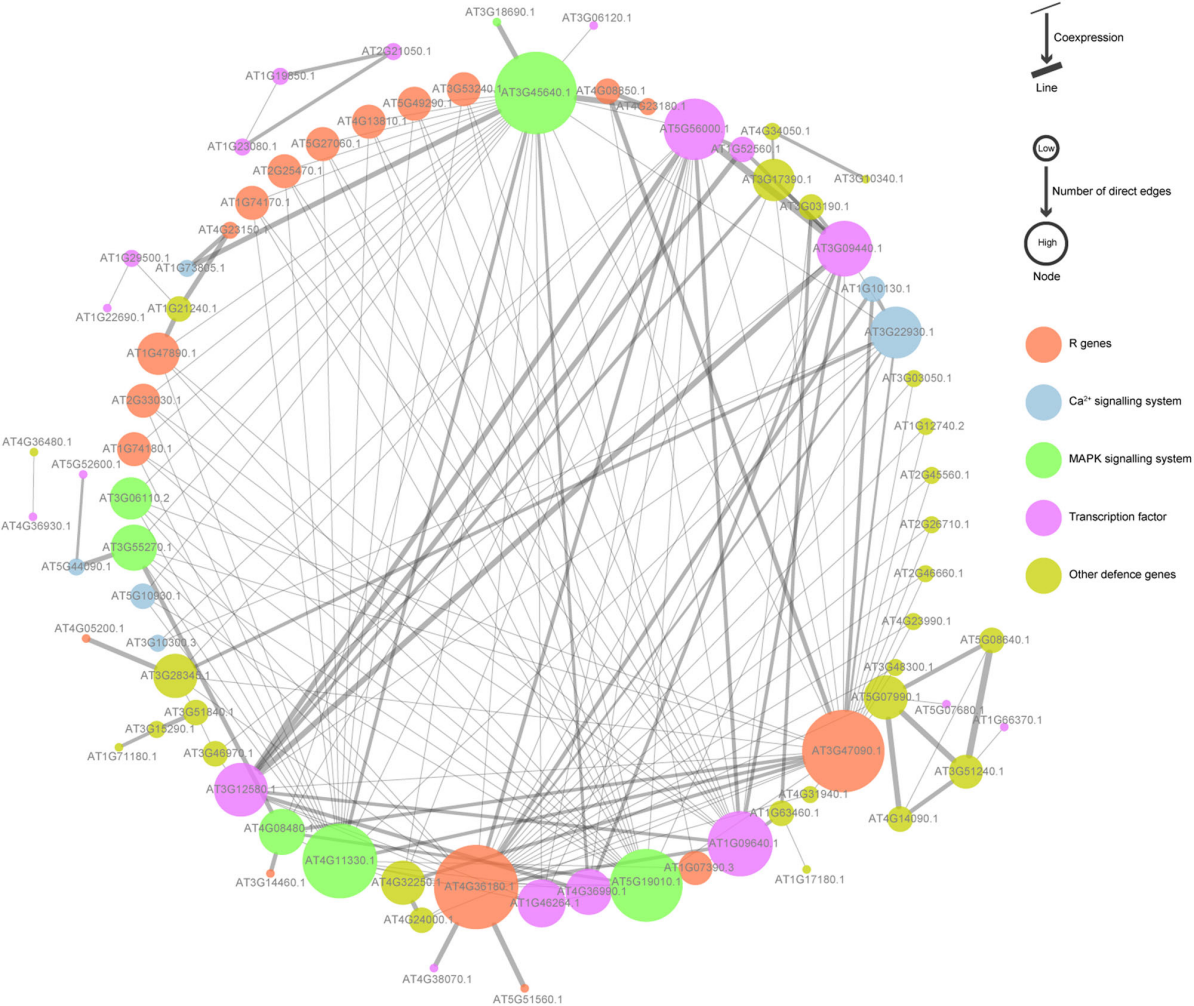
Interaction analysis of putative DEGs that are associated with disease resistance and are involved in *Ca. sinensis* defence to *C. fructicola*. The analysis was generated with a PPI network of *Arabidopsis thaliana*

To explore the possible signal pathway in tea plant defence to *C. fructicola*, the putative 88 key DEGs specific to the resistant cultivar (ZC108) and 60 shared DEGs were used to build a PPI network with Arabidopsis. A total of 126 unique TAIR IDs from the putative 148 genes were identified, and 76 of these TAIR IDs directly interacted with each other. The network analysis was used for treating the network as directed; the average number of direct neighbours in the network for each gene was 4.974. In this network, MAPKs; Ca^2+^ signalling; TFs; and pathogen receptors (resistance genes, R genes), likely RLKs, RLPs, cysteine-rich RLKs and NBS-LRRs, were significantly correlated. Seven hub nodes (MAPK) were involved in tea plant defence against *C. fructicola*, which suggests that the MAPK signalling pathway is the crucial signal transduction pathway; R genes were the major pathogen receptors to *C. fructicola* stimulation, as 18 nodes interacted with major signal pathway and defence genes (Fig. 5).

### HR and H_2_O_2_ accumulation observation in inoculated leaves

**Fig. 6:**
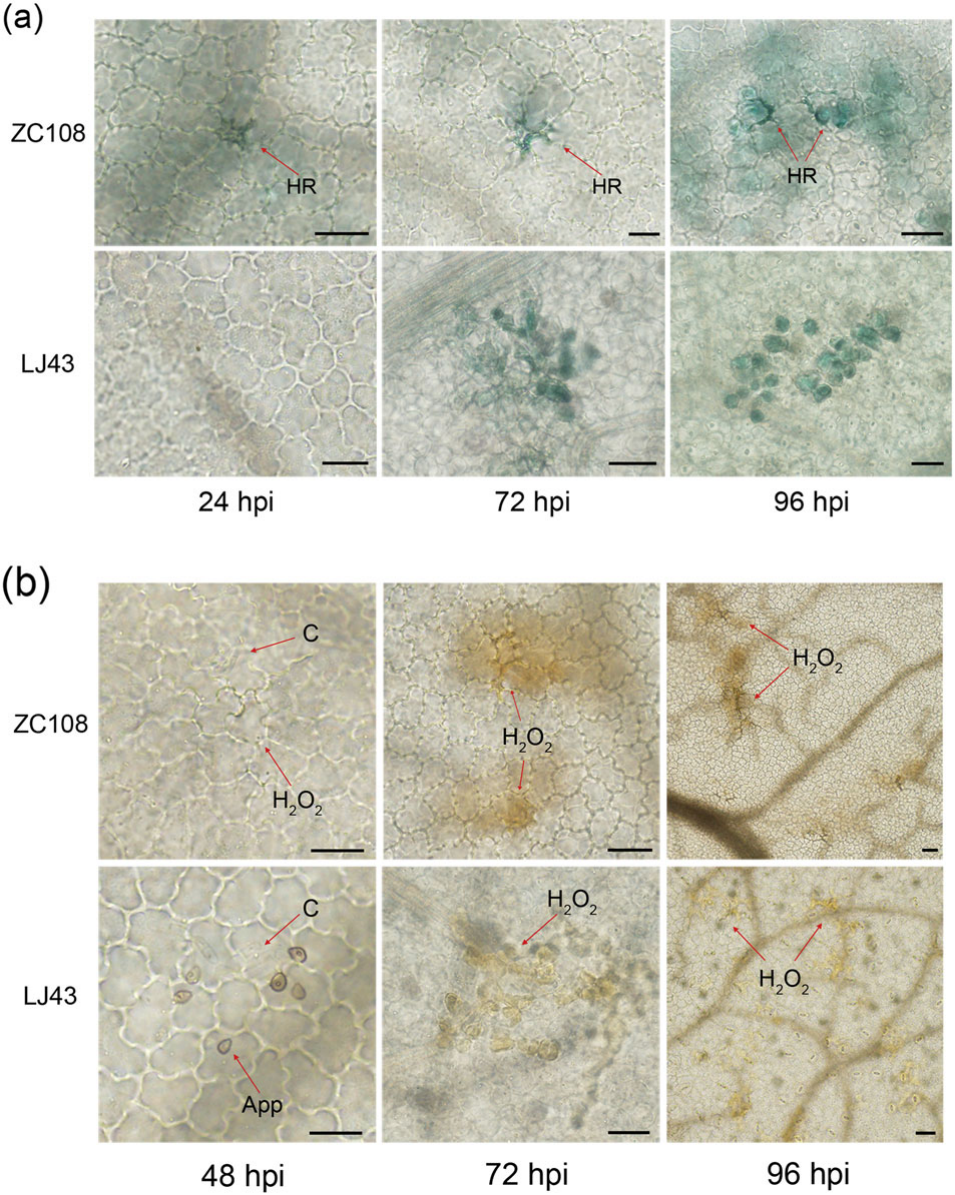
Microscopic detections of the HR and H_2_O_2_ accumulation in the leaves of ZC108 and LJ43 following *C. fructicola* infection at different times. **a** Trypan blue staining showing HR lesions in the leaves of ZC108 following *C*. *fructicola* infection; **b** H_2_O_2_ accumulation in the leaves of ZC108 and LJ43 visualised by DAB staining. C conidia, App appressorium, HR hypersensitive response. Scale bars: 10 μm

To confirm whether the HR and H_2_O_2_ are involved in tea plant defence to *C. fructicola*, the leaves of ZC108 and LJ43 were inoculated with conidial suspensions and then stained with trypan blue and DAB solution, respectively. As shown in Fig. 6a, ZC108 leaves exhibited clear HR generation during *C. fructicola* infection, whereas LJ43 leaves did not. Also, the ZC108 leaves exhibited thickening of the cell walls and epidermal cell necrosis at 24 hpi; these symptoms gradually increased as infection time increased (72 and 96 hpi), and mesophyll cells became necrotic. In contrast, mesophyll cell necrosis was observed only in LJ43 leaves at 72 and 96 hpi, but the epidermal cells were not necrotic. These results suggest that hyphae successfully infected LJ43 leaves in vivo but did not elicit a HR. Therefore, the HR is associated with *Ca. sinensis* resistance to *C. fructicola*.

Microscopic analysis indicated that brown precipitate occurred more on the ZC108 leaves than on the LJ43 leaves. The epidermal cells of ZC108 leaves exhibited H_2_O_2_ accumulation and cell wall thickening, but H_2_O_2_ accumulation was observed only in the mesophyll cells of LJ43 (Fig. 6b). Together, these results illustrated that the HR and H_2_O_2_ accumulation are involved in the resistance of tea plant against *C. fructicola* infection, and the structure of the cell wall in tea plant cells could play an important role in the defence against the pathogen.

### Validation of differential expression data

To validate the RNA-Seq results, eight DEGs that were randomly selected from RNA-Seq data were analysed using qRT-PCR. The expression results showed that the expression patterns were similar between the qRT-PCR and RNA-Seq data at different times post inoculation, suggesting reliable expression data by RNA-Seq (Supplementary Fig. S4).

## Discussion

Anthracnose causes severe damage to tea production. However, little is known about the molecular mechanisms of tea plant against *Colletotrichum*. In this study, we used RNA-Seq to analyse the upregulated DEGs in a resistant tea plant cultivar during the different stages of the *C. fructicola* infection process. The experimental results revealed that the HR and H_2_O_2_ play crucial roles in disease resistance; these defence responses might be mediated by multiple R genes, Ca^2+^ signalling and MAPK signalling; at the same time, sugar metabolism and secondary metabolites are also involved in tea plant defence to anthracnose.

### R genes specifically recognise pathogenic secretions

At the initial stage of infection, *Colletotrichum* species secrete various virulence factors into host cells to facilitate successful invasion. With respect to the plant response, R genes are responsible for specifically recognising pathogenic secretions, and these complexes trigger the plant immune system^12,30,31,32^. In our study, 23 R genes (7 *NBS-LRR*s and 16 *PRR*s) specific to the resistant tea plant as well as 15 genes shared between the resistant and susceptible cultivars were identified, and the expression of these genes was significantly induced by *C. fructicola* (Supplementary Table S6, S7 and Fig. 4b). Jayaswall et al.^16^ reported that 25 *NBS-LRR*s are also involved in tea plant defence against *E. vexans* using RNA-Seq. These findings suggest that multiple R genes are involved in tea plant defence. In general, pathogen effectors can disturb or inhibit PAMP-PRR complex-activated defence signalling networks for successful infection^33–36^, and NBS-LRRs can sequester effectors and reactivate the plant immune system^37,38^. Interestingly, we observed that these 23 R genes were significantly expressed in the resistant tea plant but were not expressed in the susceptible cultivar. Therefore, we hypothesised that *Colletotrichum* effectors may inhibit the PAMP-PRR complexes in the susceptible cultivar, resulting in the successful invasion. On the other hand, seven *NBS-LRR* genes may specifically recognise the related effectors in the resistant cultivar and activate downstream signalling to reactivate the innate immunity to restrict *Colletotrichum* infection.

### MAPK, Ca^2+^ signalling pathways are meditated by R gene and triggers PCD

MAPK cascades, which consist of MAPKKK, MAPKK and MAPK, are among the most important signalling pathways and are activated by R genes^39^. In our study, we obtained seven MAPK genes that were significantly induced by *C. fructicola*, and the PPI network showed that these candidate MAPK and R genes can directly interact with each other; meanwhile, seven Ca^2+^ signalling-related genes that were upreregulated and associated with plant defence were identified^40^, but only four of these genes interacted with R genes and other defence-related genes in the PPI network (Supplementary Table S7, and Figs. 4b and 5). These results demonstrated that the MAPK and Ca^2+^ signalling may be mediated by R genes following *Colletotrichum* infection, and MAPK cascades constitute the main defence signalling pathway. In addition, the expression of *MAPK5* was significantly upregulated in the resistant tea plant cultivar following *C. fructicola* infection but was downregulated in the susceptible cultivar (Fig. 4b). Interestingly, the proteome data of Li et al.^41^ indicated that the expression of MAPK5 is downregulated in susceptible wheat leaves after *Blumeria graminis* f. sp. *tritic* infection. These results suggest that the specific upregulated expression of MAPK5 may play an important role in tea plant defence against *C. fructicola*. In addition, *MAPK phosphatase 2* (*MKP2*) can regulate MAPK signalling and cell death to enhance Arabidopsis defence against biotrophic and necrotrophic pathogens by regulating *MAPK3* and *MAPK6*^42,43^. We observed that *MKP2* and *MAPK3* expression also clearly increased in resistant tea plant cultivars after *C. fructicola* infection. On the other hand, only one unigene of *MAPK6* was identified, and its expression was lower. Moreover, the results of the GO analysis demonstrated that PCD was also a defensive pathway that contained enriched DEGs in the resistant cultivar (Fig. 3c), and microscopic observations also showed that the HR significantly accumulated around the *C. fructicola* hyphal infection site in the resistant tea plant cultivar (Fig. 6). These results were similar to those of Vilela et al.^43^. Furthermore, we observed the upregulated expression of three genes associated with PCD in the resistant tea plant: two *Development and Cell Deaths* and one *Long Chain Base Biosynthesis Protein 1* (Fig. 4b)^44,45^. We therefore speculated that *MKP2* may regulate *MAPK3* and *MAPK5*, and positively activate the three genes associated with PCD in tea plant in defence against *C. fructicola*.

### ROS bursts regulate multiple defence responses

In general, ROS bursts constitute one of the earliest plant responses to pathogen invasion. As a signalling molecule, ROS can regulate PCD in plants during pathogen infection and can mutually regulate MAPK signalling^39,46,47^. Senthilkumar et al.^14^ reported that the HR and ROS bursts are involved in tea plant defence against *P. theae*. In our study, we observed that DEGs were enriched in the H_2_O_2_ catabolic process (Fig. 3c), and the histochemistry results indicated that H_2_O_2_ levels are significantly higher in the resistant cultivar than in the susceptible following *C. fructicola* infection (Fig. 6b). These results showed that H_2_O_2_ plays an important role in tea plant defence against multiple diseases, including anthracnose, and H_2_O_2_ production may be regulated by MAPK signalling. Li et al.^48^ recently reported that a C2H2-type TF can affect H_2_O_2_ levels by suppressing peroxidase to enhance the broad-spectrum blast resistance of rice. We discovered that *peroxidase 2* (*PA2*) (TR123656|c4_g4), which is clearly induced by *C. coccodes* in *Capsicum annuum*^49^, is enriched in H_2_O_2_ catabolic processes and that the expression of this gene is distinctly upregulated in the susceptible tea plant cultivar but not in the resistant cultivar (Fig. 4b)^50^. Hence, we suggested that *PA2* may be a negative regulator of H_2_O_2_ production and that *PA2* is suppressed by an unknown TF to increase H_2_O_2_ accumulation in the resistant tea plant; in turn, this increased H_2_O_2_ accumulation limits *Colletotrichum* infection. ROS metabolism, which is localised in the peroxisome, is usually controlled by the protein peroxin 11a (PEX11a) under stress conditions^51^. Notably, we observed that PEX11a was distinctly upregulated in the resistant tea plant during *C. fructicola* infection and may also be associated with H_2_O_2_ production. In addition, thickening of cell walls, which constitute an important of physical barrier to invading pathogens, is induced by H_2_O_2_ generation and is associated with *wall-associated kinase 3*^52,53^. In the present study, the cell walls were significantly reinforced at the penetration sites of *C. fructicola* hyphae, and this reinforcement was accompanied by H_2_O_2_ accumulation in both epidermal and mesophyll cells. At the same time, one *wall-associated kinase 3*, a cell signalling receptor, was upregulated in the resistant cultivar; on the other hand, H_2_O_2_ was generated only in mesophyll cells in the susceptible cultivar. These results suggested that H_2_O_2_ may regulate cell wall strengthening and activate signalling to resist *C. fructicola* attack. Overall, we suggested that H_2_O_2_ plays a significant role in tea plant defence to *C. fructicola*, H_2_O_2_ generation may be directed by PEX11a under pathogen stress and mediated by MAPK cascades, and PA2 may be inhibited by other genes to maintain higher levels of H_2_O_2_ in tea plant to defend against *C. fructicola*. Therefore, the function of ROS during the interaction between tea plant and *Colletotrichum* needs further clarification.

### Multiple metabolic pathways are involved in disease resistance

During plant interactions with pathogens, carbohydrate metabolism increases in the host not only to supply massive energy to defence responses but also to regulate the expression of resistance-related genes^54^. More DEGs were enriched in carbohydrate metabolism in the resistant tea plant cultivar than in the susceptible tea plant cultivar following *C. fructicola* infection (Figs. 2b and 3c), and the expression of six specific genes associated with carbohydrate metabolism was significantly upregulated (Fig. 4b). Among these genes, *6-phosphogluconate dehydrogenase*, which encodes a protein positively induced by powdery mildew in wheat leaves, has been reported to activate host defences^41^, and three *UDP-glycosyltransferase* genes that are closely related to the mechanism of galloylated catechins and flavonol 3-*O*-guycosides in tea were induced^55^. We previously demonstrated that the content of (-)-epigallocatechin-3-gallate and caffeine rapidly accumulated after *C. fructicola* infection; at the same time, the expression levels of key genes associated with flavonoids and the caffeine metabolism pathway were clearly upregulated, including the phenylalanine ammonia-lyase (*PAL*) and *S*-adenosylmethionine synthetase (*SAMS*)^4^. In the present study, DEGs involved with phenylpropanoid and its downstream metabolism of flavonoids were enriched in the resistant tea plant, and the expression levels of *PAL* (TR106249|c2_g2) and *SAMS* (TR316985|c0_g1) also increased by *C. fructicola* (Fig. 4b). These results were similar to those of Figueiredo et al.^56^, in which *SAMS* and *PAL* were involved in grapevine species defence against multiple pathogen attack. In general, sugars, which are sources of carbon, are mainly acquired by pathogens from their hosts^57^. Based on their biosynthesis and transference, fatty acids have recently been reported to be important organic nutrients between microbes and hosts; RAM2 and ATP-binding cassette transporters play critical roles in these processes^58^. Our results showed that eight genes associated with the fatty acid metabolism were detected, and the expression of these genes was higher in the resistant tea plant than in the susceptible tea plant cultivar. In contrast, these genes were either downregulated or not regulated in the susceptible cultivar (Fig. 4b). Despite these results being similar to those of Jiang et al.^58^, the role of fatty acids in the interaction between tea plant and *Colletotrichum* needs to be further explained^16,59^.

### Plant hormones associated with tea plant defence to *Colletotrichum*

In addition, plant hormones play important roles in plant defence against pathogens. In our study, the shared genes in the two cultivars were enriched in various phytohormone-related pathways, including responses to ethylene, cytokinin and auxin (Fig. 2b). Meanwhile, six related genes (*ethylene response factor 1*, *like AUXIN RESISTANT 2*, one auxin-responsive protein family gene, two gibberellin-regulated family genes, and one SAUR-like auxin-responsive protein family gene) were induced by *C. fructicola* (Supplementary Table S6). At the same time, the expression levels of six key genes that are specific to the resistant tea plant increased, including two gibberellin-regulated family gene, one auxin efflux carrier family gene, two SAUR-like auxin-responsive protein family gene and one ethylene-responsive element binding protein-coding gene (Supplementary Table S7), suggesting that these genes are involved in the regulation of plant hormones to activate plant defence responses^60,61^.

## Conclusion

**Fig. 7:**
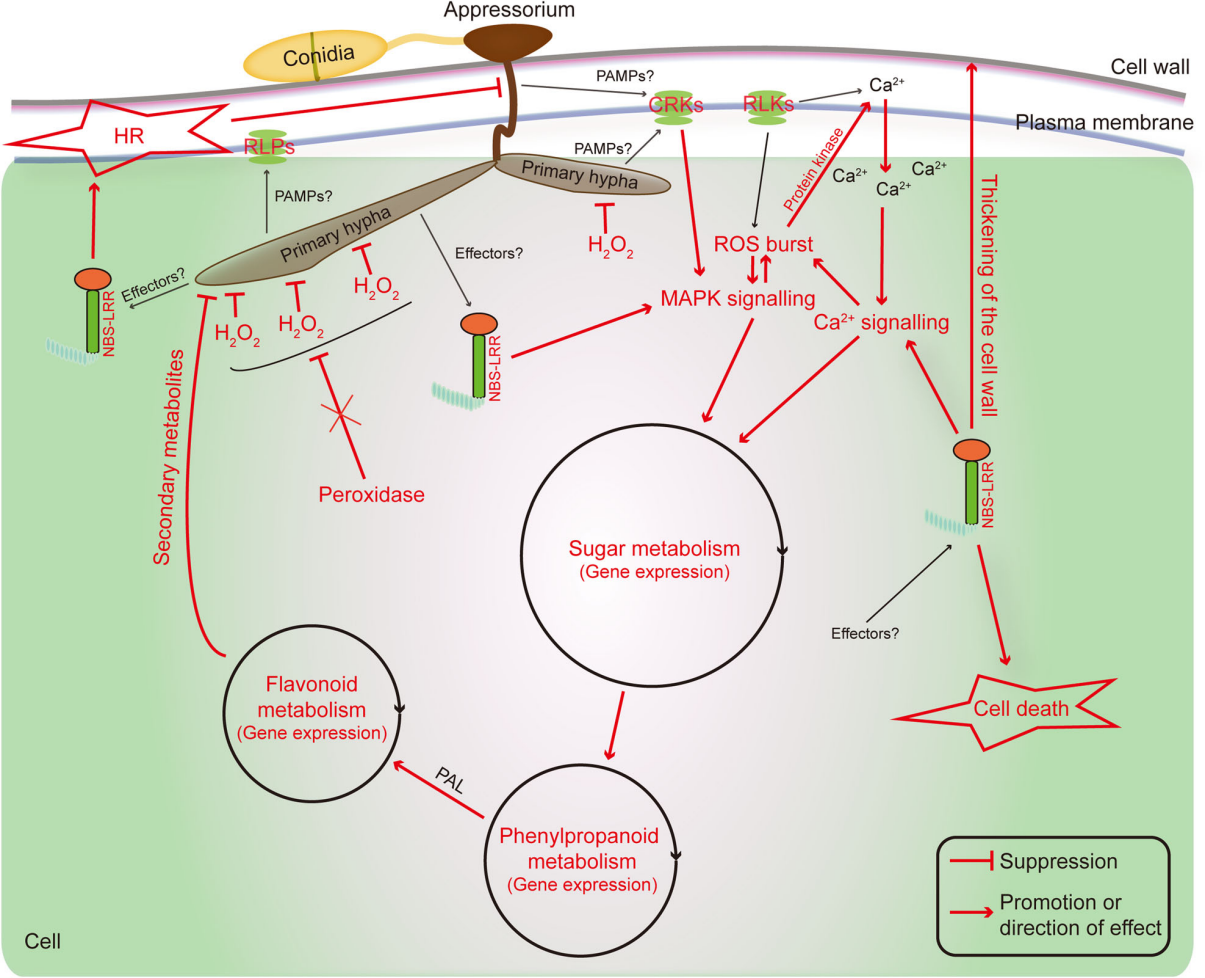
Hypothetical model for *Ca*. *sinensis* defence against anthracnose based on the results of our study. The defence function is shown in red

Tea plant has a complex defence network to defend against various pathogens. We constructed a possible model of *Ca. sinensis* defence against anthracnose based on the results of our study (Fig. 7). During the early stage of infection, *Colletotrichum* appressoria successfully penetrate the cells of tea plant. The formed primary hyphae then secrete and transport diverse virulence factors into the cell, and the pathogen receptors (RLKs, RLPs and NBS-LRRs) specifically recognise pathogenic secretions in the host. The activated pathogen receptors subsequently trigger the defence signalling, which includes MAPK and Ca^2+^ signalling pathways. At the same time, the ROS (H_2_O_2_) production is regulated by these two signalling pathways. Activated R genes also regulate the thickening of cell wall tissue to defend against hyphal growth. The activated signalling pathways trigger the expression of genes associated with the interdependency metabolism pathway, including those involved with sugar, phenylpropanoid, flavonoids and lipid metabolisms; these metabolites, most likely EGCG, suppress the invading pathogen. The activated R genes also trigger a sustained induction of both MAPK and Ca^2+^ signalling as well as the continuous production of H_2_O_2_ in the peroxisome. This signalling and production regulate HR-associated cell death and H_2_O_2_ accumulation around the hyphal infection sites. Furthermore, the functional elimination of peroxidase was suppressed by an unknown factor, extending the H_2_O_2_ inhibition (Fig. 7).

## Electronic supplementary material

Supplementary information

Supplementary information
