# Supplementary material for: Transcriptional analysis and histochemistry reveal that hypersensitive cell death and H2O2 have crucial roles in the resistance of tea plant (Camellia sinensis (L.) O. Kuntze) to anthracnose
Source: Hortic Res. 2018 Apr 1;5:18. doi: 10.1038/s41438-018-0025-2 (PMC5878829; doi:10.1038/s41438-018-0025-2)
Supplement: Supplementary file 1 — Supplementary information [file 41438_2018_25_MOESM1_ESM.docx]

**Supplementary Information**

**Transcriptional analysis and histochemistry reveal that hypersensitive cell death and H_2_O_2_ have crucial roles in the resistance of tea plant (*Camellia sinensis* (L.) O. Kuntze) to anthracnose**

Yuchun Wang^1^*, Xinyuan Hao^1^*, Qinhua Lu^1^, Lu Wang^1^, Wenjun Qian^1^, Nana Li^1^, Changqing Ding^1^, Xinchao Wang^1^ and Yajun Yang^1^

^1^ Tea Research Institute, Chinese Academy of Agricultural Sciences/National Center for Tea Improvement/Key Laboratory of Tea Biology and Resources Utilization, Ministry of Agriculture, Hangzhou 310008, People’s Republic of China

**Author for correspondence:**

*Xinchao Wang*

*Tel/Fax:* *+86 571-8665 316*

*Email: xcw75@tricaas.com*

*Yajun Yang*

*Tel/Fax: +86 571-8665 0226*

*Email: yjyang@tricaas.com*

***These authors contributed equally to this work.**


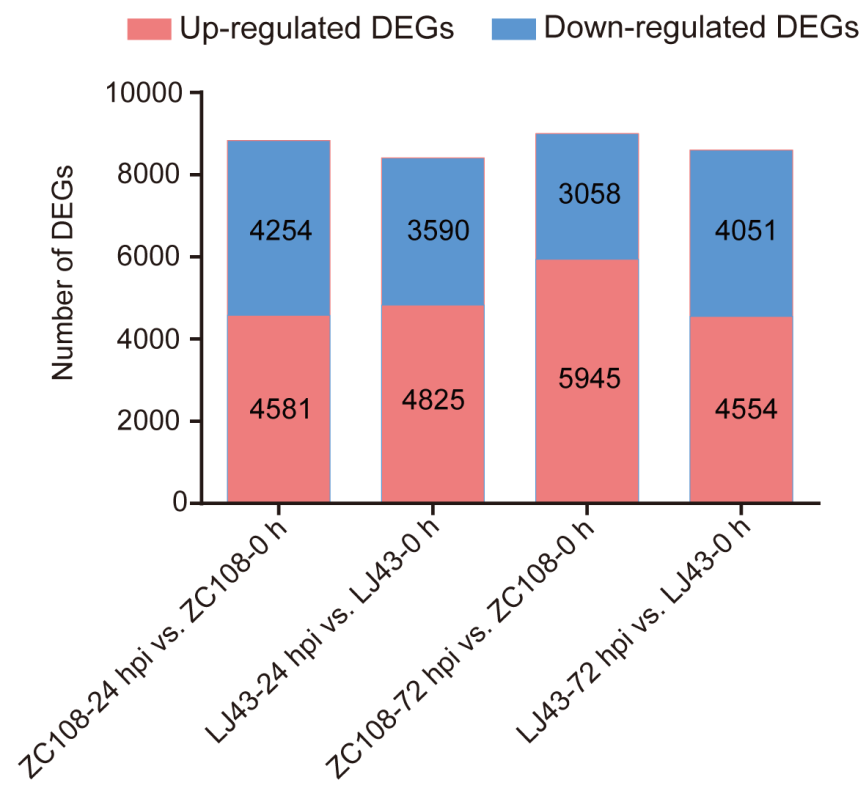


**Supplementary** **Fig.** **S1** Number of up- and down-regulated genes in ZC108 and LJ43 following inoculation with *C. fructicola*.


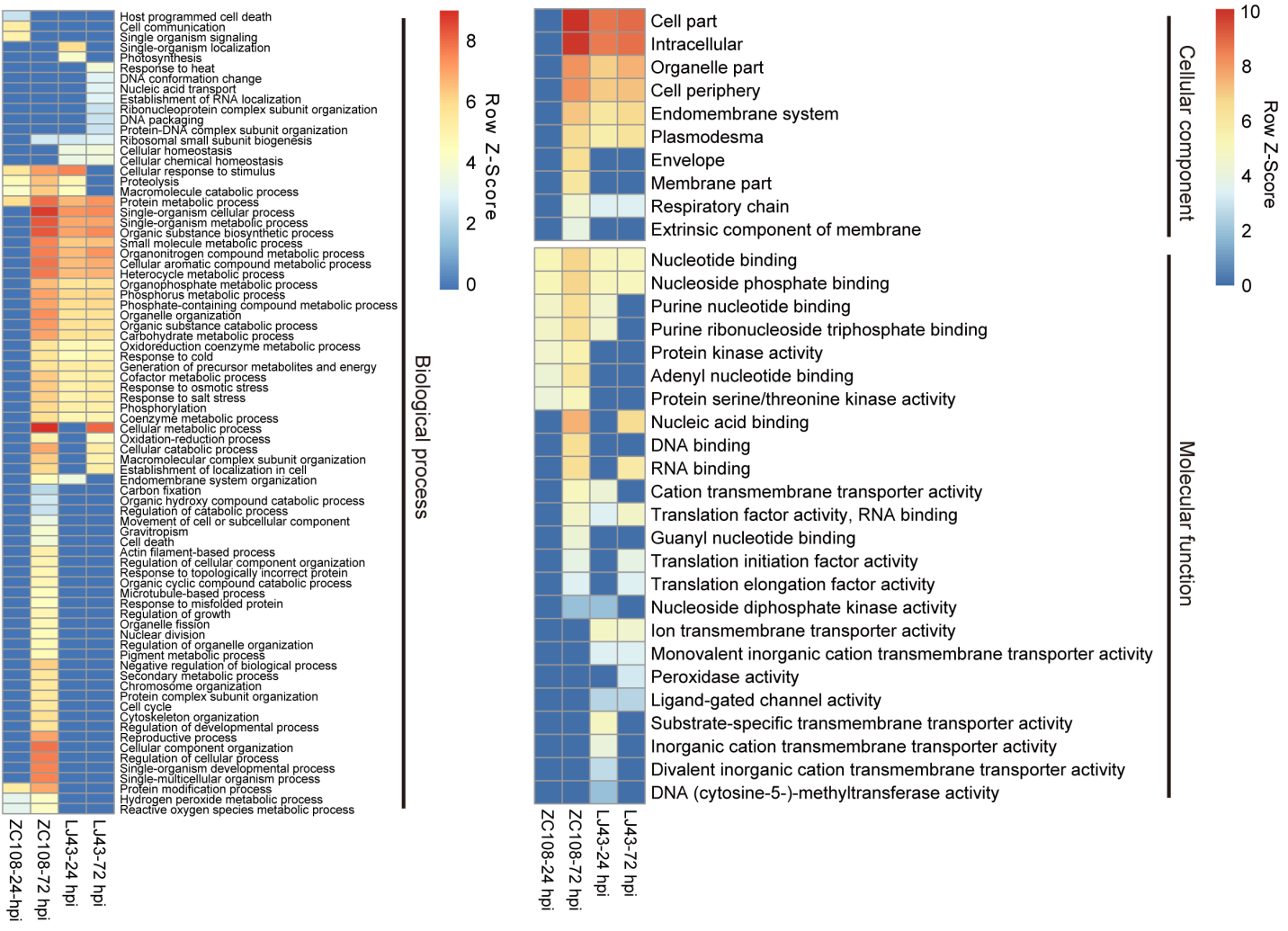


**Supplementary Fig. S2** Heatmap of enriched GO terms identified from up-regulated DEGs in resistant (ZC108) and susceptible (LJ43) *Ca. sinensis* cultivars. Terms are considered enriched when *P* < 0.05.

**
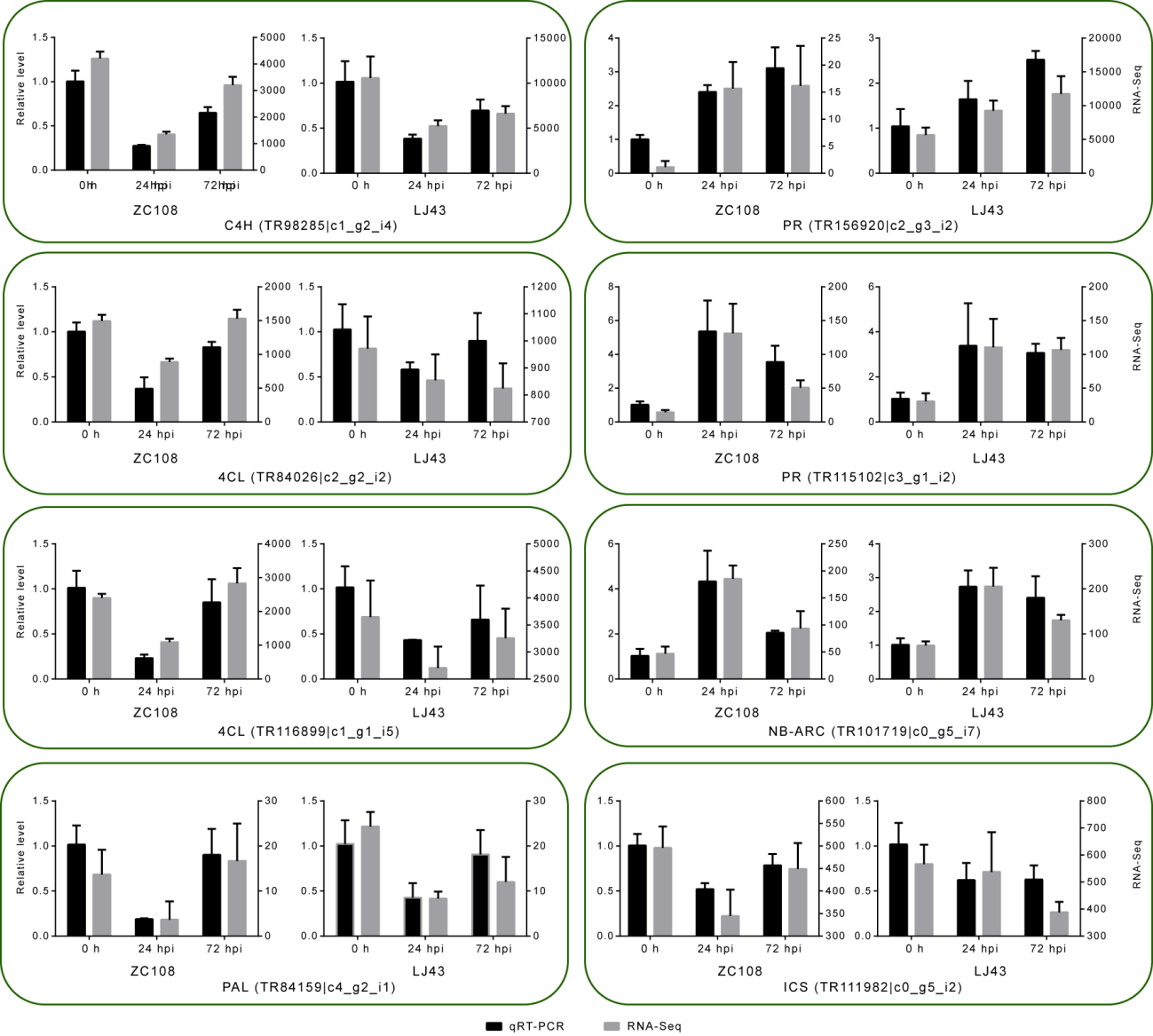
**

**Supplementary** **Fig. S3** Expression profile comparisons between the RNA-Seq and qRT-PCR data. C4H: cinnamic acid 4-hydroxylase; PR: pathogenesis-related protein, 4CL: 4-coumarate-CoA ligase; NB-ARC: nucleotide-binding adaptor shared by APAF-1, R proteins, and CED-4; PAL: phenylalanine ammonia-lyase; ICS: isochorismate synthase.

**Supplementary Table S1 Primers used in the study**

| Unigene | Primer | Sequence (5’→3’) |
| --- | --- | --- |
| TR84159\|c4_g2_i1 | Forward | CAAGGCCTTACATGGTGGCAATTTCCAAGG |
|  | Reverse | GGCAAACATGAGCTTTCCTATCGCTGCTAC |
| TR98285\|c1_g2_i4 | Forward | CTACCTCCAAGCTGTAGTCAAAGAGACCCT |
|  | Reverse | CCTGAACTCTTCTGGGTTCTTCCAGTTGTC |
| TR84026\|c2_g2_i2 | Forward | CTCCACAATTCTCCCGAATTCGTGTTCTCC |
|  | Reverse | GCTTGTTTTTCGATCTCCGCAGGAGTGTAG |
| TR116899\|c1_g1_i5 | Forward | GAGTGTAGAATGGGTTGGCAGTGGTAGTGA |
|  | Reverse | CCGGTAAATCCTACTCTTTCTCCGAAACCC |
| TR156920\|c2_g3_i2 | Forward | CTTGGTTCATCCCTCCTATGCCCAAAACTC |
|  | Reverse | CATAAGGCCCACGAGAGTGGATGAGATTG |
| TR115102\|c3_g1_i2 | Forward | CTGGGGCTCAAGTAACAGTGAGGATAGTTG |
|  | Reverse | CTGGTAATTGACTATGAGGTGGCCTTGAGC |
| TR101719\|c0_g5_i7 | Forward | GATGTATGGGAGGAAGAAACAGTGGTGG |
|  | Reverse | CCTAATCCAGGCATTCCGACAATCGAG |
| TR111982\|c0_g5_i2 | Forward | GAGGAACCTCTTAATGGAGCGCCAATCATC |
|  | Reverse | CACTACCAATGGGATTGGGCATGGAAATGG |

**Supplementary Table S2 The quality of RNA-seq**

| Cultivar | Time | The biological replicates | Raw reads | Clean reads | Raw bases (G) | Clean bases (G) | Error (%) | Q20 (%) | Q30 (%) | GC (%) |
| --- | --- | --- | --- | --- | --- | --- | --- | --- | --- | --- |
| LJ43 | 0 h | 1 | 50945750 | 49170739 | 15.28 | 14.75 | 0.03 | 96.25 | 92.03 | 46.56 |
|  |  | 2 | 46384760 | 44422449 | 13.92 | 13.33 | 0.04 | 95.39 | 89.40 | 45.78 |
|  |  | 3 | 43563977 | 42549769 | 13.07 | 12.76 | 0.03 | 95.84 | 90.09 | 47.25 |
|  | 24 h | 1 | 47480926 | 46068908 | 14.24 | 13.82 | 0.03 | 96.08 | 90.79 | 46.78 |
|  |  | 2 | 52849264 | 51403342 | 15.81 | 15.38 | 0.03 | 96.38 | 92.28 | 46.75 |
|  |  | 3 | 53416709 | 51431835 | 15.98 | 15.39 | 0.03 | 96.41 | 92.36 | 46.91 |
|  | 72 h | 1 | 44188554 | 42891007 | 13.22 | 12.84 | 0.03 | 96.07 | 91.78 | 45.86 |
|  |  | 2 | 48419428 | 46644926 | 14.49 | 13.96 | 0.03 | 96.34 | 92.22 | 46.28 |
|  |  | 3 | 40766386 | 39251002 | 12.2 | 11.75 | 0.03 | 96.44 | 92.36 | 46.32 |
| ZC108 | 0 h | 1 | 43622671 | 42584522 | 13.09 | 12.78 | 0.03 | 95.77 | 89.98 | 46.88 |
|  |  | 2 | 42910404 | 41437668 | 12.84 | 12.4 | 0.03 | 96.43 | 92.33 | 47.14 |
|  |  | 3 | 53782008 | 51537954 | 16.1 | 15.42 | 0.03 | 96.59 | 92.62 | 46.48 |
|  | 24 h | 1 | 46436452 | 45543037 | 13.93 | 13.66 | 0.03 | 96.08 | 90.56 | 46.73 |
|  |  | 2 | 47003011 | 45989966 | 14.1 | 13.8 | 0.03 | 96.07 | 90.52 | 46.70 |
|  |  | 3 | 46101500 | 45187730 | 13.83 | 13.56 | 0.03 | 96.11 | 90.52 | 46.59 |
|  | 72 h | 1 | 50256701 | 48898354 | 15.08 | 14.67 | 0.03 | 96.17 | 90.95 | 46.97 |
|  |  | 2 | 63051545 | 61269655 | 18.92 | 18.38 | 0.03 | 95.61 | 89.98 | 46.26 |
|  |  | 3 | 57093671 | 54966543 | 17.13 | 16.49 | 0.03 | 96.54 | 91.72 | 46.94 |
|  |  | Total | 878273717 | 851249406 | 263.23 | 255.14 |  |  |  |  |

**Supplementary Table S3 Summarized results of the tea plant transcriptome**

|  | Numbers |
| --- | --- |
| All transcripts | 864790 |
| All unigenes | 497332 |
| Unigenes annotated by NR | 234496 |
| Unigenes annotated by TAIR10 | 134208 |
| Contig N50 (bp) | 788 |
| Average contig (bp) | 611 |
| Total assembled bases (bp) | 528647639 |

**Supplementary Table S4 The pattern of differentially expressed genes in ZC108 and LJ43**

|  | ZC108 | | | LJ43 | | |
| --- | --- | --- | --- | --- | --- | --- |
|  | 0 h | 24 h | 72 h | 0 h | 24 h | 72 h |
| Pattern1 | 1 | 1 | 1 | 1 | 1 | 1 |
| Pattern2 | 1 | 1 | 1 | 1 | 1 | 2 |
| Pattern3 | 1 | 1 | 1 | 1 | 2 | 1 |
| Pattern4 | 1 | 1 | 1 | 1 | 2 | 2 |
| Pattern5 | 1 | 1 | 1 | 2 | 1 | 1 |
| Pattern6 | 1 | 1 | 1 | 2 | 1 | 2 |
| Pattern7 | 1 | 1 | 1 | 2 | 2 | 1 |
| Pattern8 | 1 | 1 | 1 | 2 | 2 | 2 |
| Pattern9 | 1 | 1 | 2 | 1 | 1 | 1 |
| Pattern10 | 1 | 1 | 2 | 1 | 1 | 2 |
| Pattern11 | 1 | 1 | 2 | 1 | 2 | 1 |
| Pattern12 | 1 | 1 | 2 | 1 | 2 | 2 |
| Pattern13 | 1 | 1 | 2 | 2 | 1 | 1 |
| Pattern14 | 1 | 1 | 2 | 2 | 1 | 2 |
| Pattern15 | 1 | 1 | 2 | 2 | 2 | 1 |
| Pattern16 | 1 | 1 | 2 | 2 | 2 | 2 |
| Pattern17 | 1 | 2 | 1 | 1 | 1 | 1 |
| Pattern18 | 1 | 2 | 1 | 1 | 1 | 2 |
| Pattern19 | 1 | 2 | 1 | 1 | 2 | 1 |
| Pattern20 | 1 | 2 | 1 | 1 | 2 | 2 |
| Pattern21 | 1 | 2 | 1 | 2 | 1 | 1 |
| Pattern22 | 1 | 2 | 1 | 2 | 1 | 2 |
| Pattern23 | 1 | 2 | 1 | 2 | 2 | 1 |
| Pattern24 | 1 | 2 | 1 | 2 | 2 | 2 |
| Pattern25 | 1 | 2 | 2 | 1 | 1 | 1 |
| Pattern26 | 1 | 2 | 2 | 1 | 1 | 2 |
| Pattern27 | 1 | 2 | 2 | 1 | 2 | 1 |
| Pattern28 | 1 | 2 | 2 | 1 | 2 | 2 |
| Pattern29 | 1 | 2 | 2 | 2 | 1 | 1 |
| Pattern30 | 1 | 2 | 2 | 2 | 1 | 2 |
| Pattern31 | 1 | 2 | 2 | 2 | 2 | 1 |
| Pattern32 | 1 | 2 | 2 | 2 | 2 | 2 |
| Pattern33 | 1 | 1 | 1 | 1 | 2 | 3 |
| Pattern34 | 1 | 1 | 1 | 2 | 1 | 3 |
| Pattern35 | 1 | 1 | 1 | 2 | 2 | 3 |
| Pattern36 | 1 | 1 | 1 | 2 | 3 | 1 |
| Pattern37 | 1 | 1 | 1 | 2 | 3 | 2 |
| Pattern38 | 1 | 1 | 1 | 2 | 3 | 3 |
| Pattern39 | 1 | 1 | 2 | 1 | 1 | 3 |
| Pattern40 | 1 | 1 | 2 | 1 | 2 | 3 |
| Pattern41 | 1 | 1 | 2 | 1 | 3 | 1 |
| Pattern42 | 1 | 1 | 2 | 1 | 3 | 2 |
| Pattern43 | 1 | 1 | 2 | 1 | 3 | 3 |
| Pattern44 | 1 | 1 | 2 | 2 | 1 | 3 |
| Pattern45 | 1 | 1 | 2 | 2 | 2 | 3 |
| Pattern46 | 1 | 1 | 2 | 2 | 3 | 1 |
| Pattern47 | 1 | 1 | 2 | 2 | 3 | 2 |
| Pattern48 | 1 | 1 | 2 | 2 | 3 | 3 |
| Pattern49 | 1 | 1 | 2 | 3 | 1 | 1 |
| Pattern50 | 1 | 1 | 2 | 3 | 1 | 2 |
| Pattern51 | 1 | 1 | 2 | 3 | 1 | 3 |
| Pattern52 | 1 | 1 | 2 | 3 | 2 | 1 |
| Pattern53 | 1 | 1 | 2 | 3 | 2 | 2 |
| Pattern54 | 1 | 1 | 2 | 3 | 2 | 3 |
| Pattern55 | 1 | 1 | 2 | 3 | 3 | 1 |
| Pattern56 | 1 | 1 | 2 | 3 | 3 | 2 |
| Pattern57 | 1 | 1 | 2 | 3 | 3 | 3 |
| Pattern58 | 1 | 2 | 1 | 1 | 1 | 3 |
| Pattern59 | 1 | 2 | 1 | 1 | 2 | 3 |
| Pattern60 | 1 | 2 | 1 | 1 | 3 | 1 |
| Pattern61 | 1 | 2 | 1 | 1 | 3 | 2 |
| Pattern62 | 1 | 2 | 1 | 1 | 3 | 3 |
| Pattern63 | 1 | 2 | 1 | 2 | 1 | 3 |
| Pattern64 | 1 | 2 | 1 | 2 | 2 | 3 |
| Pattern65 | 1 | 2 | 1 | 2 | 3 | 1 |
| Pattern66 | 1 | 2 | 1 | 2 | 3 | 2 |
| Pattern67 | 1 | 2 | 1 | 2 | 3 | 3 |
| Pattern68 | 1 | 2 | 1 | 3 | 1 | 1 |
| Pattern69 | 1 | 2 | 1 | 3 | 1 | 2 |
| Pattern70 | 1 | 2 | 1 | 3 | 1 | 3 |
| Pattern71 | 1 | 2 | 1 | 3 | 2 | 1 |
| Pattern72 | 1 | 2 | 1 | 3 | 2 | 2 |
| Pattern73 | 1 | 2 | 1 | 3 | 2 | 3 |
| Pattern74 | 1 | 2 | 1 | 3 | 3 | 1 |
| Pattern75 | 1 | 2 | 1 | 3 | 3 | 2 |
| Pattern76 | 1 | 2 | 1 | 3 | 3 | 3 |
| Pattern77 | 1 | 2 | 2 | 1 | 1 | 3 |
| Pattern78 | 1 | 2 | 2 | 1 | 2 | 3 |
| Pattern79 | 1 | 2 | 2 | 1 | 3 | 1 |
| Pattern80 | 1 | 2 | 2 | 1 | 3 | 2 |
| Pattern81 | 1 | 2 | 2 | 1 | 3 | 3 |
| Pattern82 | 1 | 2 | 2 | 2 | 1 | 3 |
| Pattern83 | 1 | 2 | 2 | 2 | 2 | 3 |
| Pattern84 | 1 | 2 | 2 | 2 | 3 | 1 |
| Pattern85 | 1 | 2 | 2 | 2 | 3 | 2 |
| Pattern86 | 1 | 2 | 2 | 2 | 3 | 3 |
| Pattern87 | 1 | 2 | 2 | 3 | 1 | 1 |
| Pattern88 | 1 | 2 | 2 | 3 | 1 | 2 |
| Pattern89 | 1 | 2 | 2 | 3 | 1 | 3 |
| Pattern90 | 1 | 2 | 2 | 3 | 2 | 1 |
| Pattern91 | 1 | 2 | 2 | 3 | 2 | 2 |
| Pattern92 | 1 | 2 | 2 | 3 | 2 | 3 |
| Pattern93 | 1 | 2 | 2 | 3 | 3 | 1 |
| Pattern94 | 1 | 2 | 2 | 3 | 3 | 2 |
| Pattern95 | 1 | 2 | 2 | 3 | 3 | 3 |
| Pattern96 | 1 | 2 | 3 | 1 | 1 | 1 |
| Pattern97 | 1 | 2 | 3 | 1 | 1 | 2 |
| Pattern98 | 1 | 2 | 3 | 1 | 1 | 3 |
| Pattern99 | 1 | 2 | 3 | 1 | 2 | 1 |
| Pattern100 | 1 | 2 | 3 | 1 | 2 | 2 |
| Pattern101 | 1 | 2 | 3 | 1 | 2 | 3 |
| Pattern102 | 1 | 2 | 3 | 1 | 3 | 1 |
| Pattern103 | 1 | 2 | 3 | 1 | 3 | 2 |
| Pattern104 | 1 | 2 | 3 | 1 | 3 | 3 |
| Pattern105 | 1 | 2 | 3 | 2 | 1 | 1 |
| Pattern106 | 1 | 2 | 3 | 2 | 1 | 2 |
| Pattern107 | 1 | 2 | 3 | 2 | 1 | 3 |
| Pattern108 | 1 | 2 | 3 | 2 | 2 | 1 |
| Pattern109 | 1 | 2 | 3 | 2 | 2 | 2 |
| Pattern110 | 1 | 2 | 3 | 2 | 2 | 3 |
| Pattern111 | 1 | 2 | 3 | 2 | 3 | 1 |
| Pattern112 | 1 | 2 | 3 | 2 | 3 | 2 |
| Pattern113 | 1 | 2 | 3 | 2 | 3 | 3 |
| Pattern114 | 1 | 2 | 3 | 3 | 1 | 1 |
| Pattern115 | 1 | 2 | 3 | 3 | 1 | 2 |
| Pattern116 | 1 | 2 | 3 | 3 | 1 | 3 |
| Pattern117 | 1 | 2 | 3 | 3 | 2 | 1 |
| Pattern118 | 1 | 2 | 3 | 3 | 2 | 2 |
| Pattern119 | 1 | 2 | 3 | 3 | 2 | 3 |
| Pattern120 | 1 | 2 | 3 | 3 | 3 | 1 |
| Pattern121 | 1 | 2 | 3 | 3 | 3 | 2 |
| Pattern122 | 1 | 2 | 3 | 3 | 3 | 3 |
| Pattern123 | 1 | 1 | 1 | 2 | 3 | 4 |
| Pattern124 | 1 | 1 | 2 | 1 | 3 | 4 |
| Pattern125 | 1 | 1 | 2 | 2 | 3 | 4 |
| Pattern126 | 1 | 1 | 2 | 3 | 1 | 4 |
| Pattern127 | 1 | 1 | 2 | 3 | 2 | 4 |
| Pattern128 | 1 | 1 | 2 | 3 | 3 | 4 |
| Pattern129 | 1 | 1 | 2 | 3 | 4 | 1 |
| Pattern130 | 1 | 1 | 2 | 3 | 4 | 2 |
| Pattern131 | 1 | 1 | 2 | 3 | 4 | 3 |
| Pattern132 | 1 | 1 | 2 | 3 | 4 | 4 |
| Pattern133 | 1 | 2 | 1 | 1 | 3 | 4 |
| Pattern134 | 1 | 2 | 1 | 2 | 3 | 4 |
| Pattern135 | 1 | 2 | 1 | 3 | 1 | 4 |
| Pattern136 | 1 | 2 | 1 | 3 | 2 | 4 |
| Pattern137 | 1 | 2 | 1 | 3 | 3 | 4 |
| Pattern138 | 1 | 2 | 1 | 3 | 4 | 1 |
| Pattern139 | 1 | 2 | 1 | 3 | 4 | 2 |
| Pattern140 | 1 | 2 | 1 | 3 | 4 | 3 |
| Pattern141 | 1 | 2 | 1 | 3 | 4 | 4 |
| Pattern142 | 1 | 2 | 2 | 1 | 3 | 4 |
| Pattern143 | 1 | 2 | 2 | 2 | 3 | 4 |
| Pattern144 | 1 | 2 | 2 | 3 | 1 | 4 |
| Pattern145 | 1 | 2 | 2 | 3 | 2 | 4 |
| Pattern146 | 1 | 2 | 2 | 3 | 3 | 4 |
| Pattern147 | 1 | 2 | 2 | 3 | 4 | 1 |
| Pattern148 | 1 | 2 | 2 | 3 | 4 | 2 |
| Pattern149 | 1 | 2 | 2 | 3 | 4 | 3 |
| Pattern150 | 1 | 2 | 2 | 3 | 4 | 4 |
| Pattern151 | 1 | 2 | 3 | 1 | 1 | 4 |
| Pattern152 | 1 | 2 | 3 | 1 | 2 | 4 |
| Pattern153 | 1 | 2 | 3 | 1 | 3 | 4 |
| Pattern154 | 1 | 2 | 3 | 1 | 4 | 1 |
| Pattern155 | 1 | 2 | 3 | 1 | 4 | 2 |
| Pattern156 | 1 | 2 | 3 | 1 | 4 | 3 |
| Pattern157 | 1 | 2 | 3 | 1 | 4 | 4 |
| Pattern158 | 1 | 2 | 3 | 2 | 1 | 4 |
| Pattern159 | 1 | 2 | 3 | 2 | 2 | 4 |
| Pattern160 | 1 | 2 | 3 | 2 | 3 | 4 |
| Pattern161 | 1 | 2 | 3 | 2 | 4 | 1 |
| Pattern162 | 1 | 2 | 3 | 2 | 4 | 2 |
| Pattern163 | 1 | 2 | 3 | 2 | 4 | 3 |
| Pattern164 | 1 | 2 | 3 | 2 | 4 | 4 |
| Pattern165 | 1 | 2 | 3 | 3 | 1 | 4 |
| Pattern166 | 1 | 2 | 3 | 3 | 2 | 4 |
| Pattern167 | 1 | 2 | 3 | 3 | 3 | 4 |
| Pattern168 | 1 | 2 | 3 | 3 | 4 | 1 |
| Pattern169 | 1 | 2 | 3 | 3 | 4 | 2 |
| Pattern170 | 1 | 2 | 3 | 3 | 4 | 3 |
| Pattern171 | 1 | 2 | 3 | 3 | 4 | 4 |
| Pattern172 | 1 | 2 | 3 | 4 | 1 | 1 |
| Pattern173 | 1 | 2 | 3 | 4 | 1 | 2 |
| Pattern174 | 1 | 2 | 3 | 4 | 1 | 3 |
| Pattern175 | 1 | 2 | 3 | 4 | 1 | 4 |
| Pattern176 | 1 | 2 | 3 | 4 | 2 | 1 |
| Pattern177 | 1 | 2 | 3 | 4 | 2 | 2 |
| Pattern178 | 1 | 2 | 3 | 4 | 2 | 3 |
| Pattern179 | 1 | 2 | 3 | 4 | 2 | 4 |
| Pattern180 | 1 | 2 | 3 | 4 | 3 | 1 |
| Pattern181 | 1 | 2 | 3 | 4 | 3 | 2 |
| Pattern182 | 1 | 2 | 3 | 4 | 3 | 3 |
| Pattern183 | 1 | 2 | 3 | 4 | 3 | 4 |
| Pattern184 | 1 | 2 | 3 | 4 | 4 | 1 |
| Pattern185 | 1 | 2 | 3 | 4 | 4 | 2 |
| Pattern186 | 1 | 2 | 3 | 4 | 4 | 3 |
| Pattern187 | 1 | 2 | 3 | 4 | 4 | 4 |
| Pattern188 | 1 | 1 | 2 | 3 | 4 | 5 |
| Pattern189 | 1 | 2 | 1 | 3 | 4 | 5 |
| Pattern190 | 1 | 2 | 2 | 3 | 4 | 5 |
| Pattern191 | 1 | 2 | 3 | 1 | 4 | 5 |
| Pattern192 | 1 | 2 | 3 | 2 | 4 | 5 |
| Pattern193 | 1 | 2 | 3 | 3 | 4 | 5 |
| Pattern194 | 1 | 2 | 3 | 4 | 1 | 5 |
| Pattern195 | 1 | 2 | 3 | 4 | 2 | 5 |
| Pattern196 | 1 | 2 | 3 | 4 | 3 | 5 |
| Pattern197 | 1 | 2 | 3 | 4 | 4 | 5 |
| Pattern198 | 1 | 2 | 3 | 4 | 5 | 1 |
| Pattern199 | 1 | 2 | 3 | 4 | 5 | 2 |
| Pattern200 | 1 | 2 | 3 | 4 | 5 | 3 |
| Pattern201 | 1 | 2 | 3 | 4 | 5 | 4 |
| Pattern202 | 1 | 2 | 3 | 4 | 5 | 5 |
| Pattern203 | 1 | 2 | 3 | 4 | 5 | 6 |
| The different numbers indicate the pattern of differentially expressed genes in the ZC108 and LJ43 during *C. fructicola* inoculation times (h); the pattern of no differentially expressed genes emphasized in red. | | | | | | |

**Supplementary Table S5 Significantly differentially expressed genes that annotated with Arabidopsis database in ZC108 and LJ43.( Supplementary Table S5 is a large table, so we submitted separately as an EXCEL)**

**Supplementary Table S6 List of enriched Gene Ontology terms identified from the 3250 up-regulated DEGs in resistant (ZC108) and susceptible (LJ43) Ca. sinensis completely (p < 0.05)**

| Ontology | GO ID | Term | Number of annotated genes with Arabidopsis |
| --- | --- | --- | --- |
| Biological process | 0044763 | Single-organism cellular process | 636 |
|  | 0044237 | Cellular metabolic process | 594 |
|  | 0050794 | Regulation of cellular process | 456 |
|  | 0044710 | Single-organism metabolic process | 435 |
|  | 0051716 | Cellular response to stimulus | 310 |
|  | 0044707 | Single-multicellular organism process | 277 |
|  | 0007154 | Cell communication | 268 |
|  | 1902578 | Single-organism localization | 258 |
|  | 0016043 | Cellular component organization | 254 |
|  | 0019222 | Regulation of metabolic process | 253 |
|  | 0044281 | Small molecule metabolic process | 243 |
|  | 0080090 | Regulation of primary metabolic process | 232 |
|  | 0044700 | Single organism signaling | 226 |
|  | 0009889 | Regulation of biosynthetic process | 217 |
|  | 0060255 | Regulation of macromolecule metabolic process | 212 |
|  | 0051171 | Regulation of nitrogen compound metabolic process | 209 |
|  | 0009725 | Response to hormone | 206 |
|  | 0010468 | Regulation of gene expression | 206 |
|  | 0043207 | Response to external biotic stimulus | 202 |
|  | 0051707 | Response to other organism | 202 |
|  | 0006793 | Phosphorus metabolic process | 182 |
|  | 0006796 | Phosphate-containing compound metabolic process | 181 |
|  | 0044248 | Cellular catabolic process | 178 |
|  | 1901575 | Organic substance catabolic process | 175 |
|  | 0036211 | Protein modification process | 169 |
|  | 0070887 | Cellular response to chemical stimulus | 159 |
|  | 0005975 | Carbohydrate metabolic process | 154 |
|  | 0009653 | Anatomical structure morphogenesis | 144 |
|  | 0048869 | Cellular developmental process | 120 |
|  | 0051649 | Establishment of localization in cell | 118 |
|  | 0006970 | Response to osmotic stress | 117 |
|  | 0033554 | Cellular response to stress | 117 |
|  | 0016310 | Phosphorylation | 115 |
|  | 0048583 | Regulation of response to stimulus | 109 |
|  | 0009651 | Response to salt stress | 106 |
|  | 0045184 | Establishment of protein localization | 104 |
|  | 0070727 | Cellular macromolecule localization | 103 |
|  | 0030154 | Cell differentiation | 103 |
|  | 0048518 | Positive regulation of biological process | 101 |
|  | 0034613 | Cellular protein localization | 99 |
|  | 0006955 | Immune response | 98 |
|  | 0097305 | Response to alcohol | 86 |
|  | 0080134 | Regulation of response to stress | 84 |
|  | 1902580 | Single-organism cellular localization | 82 |
|  | 0006508 | Proteolysis | 82 |
|  | 0031347 | Regulation of defense response | 80 |
|  | 0009617 | Response to bacterium | 80 |
|  | 0051186 | Cofactor metabolic process | 76 |
|  | 0019748 | Secondary metabolic process | 76 |
|  | 0009620 | Response to fungus | 73 |
|  | 0016049 | Cell growth | 72 |
|  | 0061024 | Membrane organization | 71 |
|  | 0021700 | Developmental maturation | 71 |
|  | 0009057 | Macromolecule catabolic process | 70 |
|  | 0009743 | Response to carbohydrate | 63 |
|  | 0009409 | Response to cold | 63 |
|  | 0010941 | Regulation of cell death | 60 |
|  | 0090627 | Plant epidermal cell differentiation | 59 |
|  | 0006732 | Coenzyme metabolic process | 57 |
|  | 0002682 | Regulation of immune system process | 56 |
|  | 0043067 | Regulation of programmed cell death | 56 |
|  | 0009723 | Response to ethylene | 55 |
|  | 0006972 | Hyperosmotic response | 48 |
|  | 0006733 | Oxidoreduction coenzyme metabolic process | 47 |
|  | 0055114 | Oxidation-reduction process | 47 |
|  | 0032879 | Regulation of localization | 47 |
|  | 0009733 | Response to auxin | 46 |
|  | 0042594 | Response to starvation | 45 |
|  | 0071214 | Cellular response to abiotic stimulus | 43 |
|  | 1901361 | Organic cyclic compound catabolic process | 42 |
|  | 0000302 | Response to reactive oxygen species | 42 |
|  | 0009744 | Response to sucrose | 36 |
|  | 0034285 | Response to disaccharide | 36 |
|  | 0010256 | Endomembrane system organization | 36 |
|  | 0051049 | Regulation of transport | 33 |
|  | 0043269 | Regulation of ion transport | 31 |
|  | 0009746 | Response to hexose | 29 |
|  | 0034284 | Response to monosaccharide | 29 |
|  | 0009750 | Response to fructose | 29 |
|  | 0006857 | Oligopeptide transport | 29 |
|  | 0015833 | Peptide transport | 29 |
|  | 0072593 | Reactive oxygen species metabolic process | 26 |
|  | 0010646 | Regulation of cell communication | 26 |
|  | 0007568 | Aging | 25 |
|  | 0023051 | Regulation of signaling | 24 |
|  | 0045730 | Respiratory burst | 24 |
|  | 0010119 | Regulation of stomatal movement | 22 |
|  | 0007031 | Peroxisome organization | 21 |
|  | 0042743 | Hydrogen peroxide metabolic process | 21 |
|  | 0008219 | Cell death | 19 |
|  | 0009735 | Response to cytokinin | 19 |
|  | 0043476 | Pigment accumulation | 18 |
|  | 0043478 | Pigment accumulation in response to UV light | 18 |
|  | 0009612 | Response to mechanical stimulus | 17 |
|  | 0009825 | Multidimensional cell growth | 17 |
|  | 0009595 | Detection of biotic stimulus | 16 |
|  | 0032880 | Regulation of protein localization | 15 |
|  | 0009624 | Response to nematode | 14 |
|  | 0046149 | Pigment catabolic process | 13 |
|  | 0042752 | Regulation of circadian rhythm | 12 |
|  | 0009608 | Response to symbiont | 12 |
|  | 0040034 | Regulation of development, heterochronic | 11 |
|  | 0007033 | Vacuole organization | 11 |
|  | 1901419 | Regulation of response to alcohol | 11 |
|  | 0022406 | Membrane docking | 8 |
|  | 0048278 | Vesicle docking | 8 |
|  | 0042939 | Tripeptide transport | 7 |
|  | 0010959 | Regulation of metal ion transport | 5 |
|  | 0010506 | Regulation of autophagy | 5 |
|  | 0010555 | Response to mannitol | 5 |
|  | 0045962 | Positive regulation of development, heterochronic | 5 |
| Cellular component | 0044464 | Cell part | 1521 |
|  | 0071944 | Cell periphery | 430 |
|  | 0012505 | Endomembrane system | 155 |
|  | 0009506 | Plasmodesma | 114 |
| Molecular function | 0003676 | Nucleic acid binding | 230 |
|  | 0003677 | DNA binding | 151 |
|  | 0000166 | Nucleotide binding | 104 |
|  | 1901265 | Nucleoside phosphate binding | 104 |
|  | 0004672 | Protein kinase activity | 93 |
|  | 0017076 | Purine nucleotide binding | 78 |
|  | 0035639 | Purine ribonucleoside triphosphate binding | 78 |
|  | 0022891 | Substrate-specific transmembrane transporter activity | 76 |
|  | 0030554 | Adenyl nucleotide binding | 64 |
|  | 0004674 | Protein serine/threonine kinase activity | 63 |
|  | 0015075 | Ion transmembrane transporter activity | 61 |
|  | 0008324 | Cation transmembrane transporter activity | 49 |
|  | 0035251 | UDP-glucosyltransferase activity | 24 |
|  | 0043565 | Sequence-specific DNA binding | 19 |
|  | 0042803 | Protein homodimerization activity | 17 |
|  | 0004888 | Transmembrane signaling receptor activity | 14 |
|  | 0038023 | Signaling receptor activity | 14 |
|  | 0080161 | Auxin transmembrane transporter activity | 10 |
|  | 0001067 | Regulatory region nucleic acid binding | 10 |
|  | 0004630 | Phospholipase D activity | 8 |
|  | 0010294 | Abscisic acid glucosyltransferase activity | 7 |
|  | 0010328 | Auxin influx transmembrane transporter activity | 6 |
|  | 0051287 | NAD binding | 5 |
|  | 0015172 | Acidic amino acid transmembrane transporter activity | 4 |
|  | 0015181 | Arginine transmembrane transporter activity | 4 |

**Supplementary Table S7 The DEGs involved in defence in ZC108 and LJ43 during *C. fructicola* infection**

| Unigene | Arabidopsis ID | Functional annotation by TAIR10 | Fold change versus respective level at 0 h | | | |
| --- | --- | --- | --- | --- | --- | --- |
|  |  |  | ZC108 | | LJ43 | |
|  |  |  | 24 hpi | 72 hpi | 24 hpi | 72 hpi |
| TR284410\|c0_g2 | AT5G20860.1 | Plant invertase/pectin methylesterase inhibitor | 23.4 | 10.4 | 4.4 | 2.7 |
| TR158297\|c2_g3 | AT2G29730.1 | UDP-glucosyl transferase 71D1 | 15.1 | 8.3 | 7.5 | 3.2 |
| TR146769\|c2_g1 | AT4G23180.1 | Receptor-like protein kinase 10 | 10.8 | 6.9 | 3.3 | 3.4 |
| TR130829\|c6_g1 | AT4G23180.1 | Receptor-like protein kinase 10 | 5.0 | 3.0 | 1.7 | 1.2 |
| TR117606\|c0_g1 | AT4G23150.1 | Receptor-like protein kinase 7 | 4.2 | 2.6 | 1.3 | 1.3 |
| TR99143\|c1_g2 | AT4G23160.1 | Receptor-like protein kinase 8 | 14.5 | 9.0 | 3.7 | 1.5 |
| TR87174\|c0_g3 | AT4G08850.1 | Leucine-rich repeat receptor-like protein kinase | 11.0 | 12.0 | 5.5 | 5.6 |
| TR95360\|c3_g15 | AT1G07390.3 | Receptor-like protein 1 | 15.3 | 9.4 | 4.8 | 4.6 |
| TR112132\|c0_g1 | AT1G74170.1 | Receptor-like protein 13 | 12.2 | 6.2 | 4.2 | 2.4 |
| TR95360\|c3_g4 | AT1G74170.1 | Receptor-like protein 13 | 20.0 | 6.6 | 4.1 | 2.1 |
| TR76774\|c1_g14 | AT2G25470.1 | Receptor-like protein 21 | 12.3 | 4.6 | 3.9 | 1.4 |
| TR262614\|c0_g1 | AT3G53240.1 | Receptor-like protein 45 | 36.0 | 14.8 | 5.3 | 2.5 |
| TR91214\|c0_g1 | AT5G27060.1 | Receptor-like protein 53 | 17.8 | 5.6 | 3.3 | 1.7 |
| TR165614\|c0_g4 | AT5G49290.1 | Receptor-like protein 56 | 12.0 | 10.8 | 4.8 | 3.7 |
| TR128186\|c3_g1 | AT5G49290.1 | Receptor-like protein 56 | 18.6 | 6.3 | 4.5 | 1.6 |
| TR77167\|c4_g6 | AT1G19430.1 | S-adenosyl-L-methionine-dependent methyltransferase | 16.3 | 10.0 | 6.9 | 3.8 |
| TR115777\|c0_g1 | AT4G05070.1 | Wound-responsive family protein | 322.6 | 38.5 | 30.6 | 5.9 |
| TR81040\|c0_g1 | AT3G23240.1 | Ethylene response factor 1 | 5.0 | 61.5 | 2.0 | 21.3 |
| TR59407\|c0_g3 | AT2G46690.1 | Auxin-responsive protein family | 4.5 | 3.9 | 1.4 | 1.4 |
| TR169029\|c0_g2 | AT4G01680.2 | MYB domain protein 55 | 6.2 | 3.8 | 2.8 | 1.6 |
| TR111321\|c0_g1 | AT1G73805.1 | Calmodulin binding protein | 10.2 | 8.8 | 2.6 | 3.7 |
| TR129798\|c3_g7 | AT4G23990.1 | Cellulose synthase like G3 | 5.5 | 4.4 | 2.6 | 1.7 |
| TR95332\|c10_g1 | AT4G23990.1 | Cellulose synthase like G3 | 7.9 | 4.6 | 1.9 | 1.4 |
| TR119388\|c2_g5 | AT1G21230.1 | Wall-associated kinase 5 | 4.1 | 2.9 | 1.1 | 1.2 |
| TR76056\|c2_g3 | AT2G26710.1 | CYP450 superfamily protein | 6.2 | 7.2 | 2.8 | 2.9 |
| TR95320\|c11_g1 | AT2G46660.1 | CYP78A6 | 16.1 | 6.3 | 5.3 | 2.9 |
| TR118524\|c1_g2 | AT4G31940.1 | CYP82C4 | 4.5 | 3.0 | 1.6 | 1.2 |
| TR275649\|c0_g1 | AT1G12740.2 | CYP87A2 | 53.0 | 45.0 | 4.2 | 2.8 |
| TR125981\|c1_g3 | AT1G05570.1 | CYP90D1 | 3.6 | 3.5 | 1.8 | 1.4 |
| TR114160\|c3_g2 | AT1G67000.1 | Protein kinase superfamily protein | 16.0 | 10.0 | 3.2 | 3.1 |
| TR103441\|c0_g1 | AT3G18690.1 | MAP kinase substrate 1 | 7.9 | 4.4 | 4.1 | 3.2 |
| TR165595\|c4_g1 | AT5G19010.1 | MAP kinase 16 | 2.4 | 2.1 | 2.1 | 1.5 |
| TR85204\|c0_g3 | AT3G45640.1 | MAP kinase 3 | 3.6 | 2.4 | 2.8 | 2.1 |
| TR332886\|c0_g2 | AT4G08480.1 | MAP kinase kinase kinase 9 | 2.0 | 1.7 | 1.5 | 1.3 |
| TR78522\|c1_g1 | AT3G55270.1 | MAP kinase phosphatase 1 | 3.0 | 1.8 | 2.5 | 1.3 |

**Supplementary Table S8 List of genes specifically activated in resistant *Ca. sinensis* in response to *C. fructicola***

| Category | Unigene | Arabidopsis ID | Function annoation |
| --- | --- | --- | --- |
| R gene | TR201164\|c0_g2 | AT5G05400.1 | LRR and NB-ARC domains-containing disease resistance protein |
|  | TR133965\|c11_g8 | AT2G33030.1 | Receptor like protein 25 |
|  | TR141977\|c0_g1 | AT1G47890.1 | Receptor like protein 7 |
|  | TR97539\|c0_g2 | AT1G74180.1 | Receptor like protein 14 |
|  | TR142796\|c0_g1 | AT5G60900.1 | Receptor-like protein kinase 1 |
|  | TR171052\|c2_g5 | AT4G23150.1 | Cysteine-rich RLK (RECEPTOR-like protein kinase) 7 |
|  | TR101379\|c32_g3 | AT4G23160.1 | Cysteine -rich RLK (RECEPTOR-like protein kinase) 8 |
|  | TR125187\|c0_g4 | AT4G23160.1 | Cysteine -rich RLK (RECEPTOR-like protein kinase) 8 |
|  | TR125573\|c0_g1 | AT4G23160.1 | Cysteine -rich RLK (RECEPTOR-like protein kinase) 8 |
|  | TR330357\|c0_g1 | AT4G23160.1 | Cysteine -rich RLK (RECEPTOR-like protein kinase) 8 |
|  | TR125805\|c0_g1 | AT3G14460.1 | LRR and NB-ARC domains-containing disease resistance protein |
|  | TR95364\|c2_g12 | AT3G14470.1 | NB-ARC domain-containing disease resistance protein |
|  | TR142558\|c7_g1 | AT3G46530.1 | NB-ARC domain-containing disease resistance protein |
|  | TR329811\|c0_g2 | AT1G67720.1 | Leucine-rich repeat protein kinase family protein |
|  | TR168255\|c1_g3 | AT3G47090.1 | Leucine-rich repeat protein kinase family protein |
|  | TR135720\|c3_g3 | AT1G59780.1 | NB-ARC domain-containing disease resistance protein |
|  | TR112981\|c1_g1 | AT1G70250.1 | Receptor serine/threonine kinase |
| Sugar metabolism | TR88306\|c0_g1 | AT3G46970.1 | Alpha-glucan phosphorylase 2 |
|  | TR117294\|c0_g1 | AT1G71180.1 | 6-phosphogluconate dehydrogenase |
|  | TR132277\|c0_g1 | AT4G15550.1 | UDP-glycosyltransferase 75D1 |
|  | TR103132\|c0_g1 | AT4G21760.1 | Beta-glucosidase 47 |
|  | TR89286\|c0_g2 | AT4G14090.1 | UDP-glycosyltransferase 75C1 |
|  | TR154048\|c0_g5 | AT1G05530.1 | UDP-glycosyltransferase 75B2 |
| Peroxidases | TR123656\|c4_g4 | AT5G06720.1 | Peroxidase 2 |
|  | TR107210\|c2_g11 | AT1G47750.1 | Peroxin 11a |
| TFs | TR133947\|c19_g4 | AT5G25150.1 | Transcription initiation factor TFIID subunit 5 |
|  | TR112295\|c4_g4 | AT4G36710.1 | GRAS family transcription factor |
|  | TR90077\|c2_g2 | AT2G46690.1 | SAUR-like auxin-responsive protein family |
|  | TR99605\|c4_g3 | AT5G07680.1 | NAC domain containing protein 4 |
|  | TR129064\|c1_g1 | AT1G74670.1 | Gibberellin-regulated family protein 6 |
|  | TR76038\|c1_g1 | AT4G36990.1 | Heat shock factor 4 |
|  | TR101972\|c1_g2 | AT3G12580.1 | Heat shock protein 70 |
| Fatty acid metabolism | TR78538\|c2_g2 | AT3G51840.1 | Acyl-CoA oxidase 4 |
|  | TR160499\|c8_g4 | AT5G43760.1 | 3-ketoacyl-CoA synthase 20 |
|  | TR116112\|c0_g1 | AT3G15290.1 | 3-hydroxyacyl-CoA dehydrogenase |
|  | TR159502\|c0_g1 | AT3G28345.1 | ABC transporter protein |
|  | TR128162\|c0_g1 | AT3G45140.1 | Lipoxygenase 2 |
|  | TR90149\|c3_g2 | AT3G45140.1 | Lipoxygenase 2 |
|  | TR99013\|c0_g1 | AT4G01660.1 | ABC transporter 1 |
| Secondary metabolism | TR106249\|c2_g2 | AT3G10340.1 | Phenylalanine ammonia-lyase 4 |
|  | TR316985\|c0_g1 | AT3G17390.1 | S-adenosylmethionine synthetase |
| CDPKs | TR132600\|c0_g2 | AT3G22930.1 | Calmodulin-like 11 |
|  | TR147489\|c2_g3 | AT3G52870.1 | IQ calmodulin-binding motif family protein |
|  | TR138793\|c1_g2 | AT3G55470.1 | Calcium-dependent lipid-binding (CaLB domain) family protein |
|  | TR268513\|c0_g2 | AT5G44090.1 | Calcium-binding EF-hand family protein |
|  | TR85794\|c0_g1 | AT1G10130.1 | Endoplasmic reticulum-type calcium-transporting ATPase 3 |
|  | TR106658\|c0_g9 | AT5G10930.1 | CBL-interacting protein kinase 5 |
| MAPKs | TR318265\|c0_g2 | AT4G11330.1 | MAP kinase 5 |
|  | TR65557\|c0_g1 | AT3G06110.2 | MAPK phosphatase 2 |
| Cell death | TR281510\|c0_g1 | AT5G01660.1 | Development and Cell Death |
|  | TR281510\|c0_g2 | AT5G01660.1 | Development and Cell Death |
|  | TR96977\|c2_g1 | AT4G36480.1 | Long chain base biosynthesis protein 1 |
| Glutathione | TR274846\|c0_g2 | AT1G63460.1 | Glutathione peroxidase 8 |
|  | TR88455\|c2_g6 | AT3G03190.1 | Glutathione S-transferase F11 |
| Others | TR152552\|c0_g1 | AT3G10220.1 | Tubulin folding cofactor B |
|  | TR286882\|c0_g1 | AT1G21240.1 | Wall associated kinase 3 |
|  | TR160752\|c0_g2 | AT4G32250.1 | Protein kinase superfamily protein |
|  | TR131131\|c0_g1 | AT4G34050.1 | S-adenosyl-L-methionine-dependent methyltransferases |
|  | TR128635\|c1_g1 | AT3G48300.1 | CYP71A23 |
|  | TR197785\|c0_g1 | AT2G45560.1 | CYP76C1 |
|  | TR94463\|c0_g1 | AT2G26710.1 | Cytochrome P450 superfamily protein |
